# Characterization of CDK(5) inhibitor, 20-223 (aka CP668863) for colorectal cancer therapy

**DOI:** 10.18632/oncotarget.23749

**Published:** 2017-12-28

**Authors:** Caroline M. Robb, Smit Kour, Jacob I. Contreras, Ekta Agarwal, Carter J. Barger, Sandeep Rana, Yogesh Sonawane, Beth K. Neilsen, Margaret Taylor, Smitha Kizhake, Rhishikesh N. Thakare, Sanjib Chowdhury, Jing Wang, Jennifer D. Black, Michael A. Hollingsworth, Michael G. Brattain, Amarnath Natarajan

**Affiliations:** ^1^ Eppley Institute for Research in Cancer, University of Nebraska Medical Center, 985950 Nebraska Medical Center, Omaha, Nebraska 68198-5950, USA; ^2^ Department of Pharmaceutical Sciences, University of Nebraska Medical Center, Omaha, Nebraska 68198-5950, USA; ^3^ Section of Gastroenterology, Department of Medicine, Boston University Medical Center, Boston, Massachusetts 02118, USA; ^4^ Fred and Pamela Buffett Cancer Center, University of Nebraska Medical Center, Omaha, Nebraska 68198-5950, USA

**Keywords:** colorectal cancer (CRC), cyclin-dependent kinase (CDK), CDK inhibitor

## Abstract

Colorectal cancer (CRC) remains one of the leading causes of cancer related deaths in the United States. Currently, there are limited therapeutic options for patients suffering from CRC, none of which focus on the cell signaling mechanisms controlled by the popular kinase family, cyclin dependent kinases (CDKs). Here we evaluate a Pfizer developed compound, CP668863, that inhibits cyclin-dependent kinase 5 (CDK5) in neurodegenerative disorders. CDK5 has been implicated in a number of cancers, most recently as an oncogene in colorectal cancers. Our lab synthesized and characterized CP668863 – now called 20-223. In our established colorectal cancer xenograft model, 20-223 reduced tumor growth and tumor weight indicating its value as a potential anti-CRC agent. We subjected 20-223 to a series of cell-free and cell-based studies to understand the mechanism of its anti-tumor effects. In our hands, *in vitro* 20-223 is most potent against CDK2 and CDK5. The clinically used CDK inhibitor AT7519 and 20-223 share the aminopyrazole core and we used it to benchmark the 20-223 potency. In CDK5 and CDK2 kinase assays, 20-223 was ∼3.5-fold and ∼65.3-fold more potent than known clinically used CDK inhibitor, AT7519, respectively. Cell-based studies examining phosphorylation of downstream substrates revealed 20-223 inhibits the kinase activity of CDK5 and CDK2 in multiple CRC cell lines. Consistent with CDK5 inhibition, 20-223 inhibited migration of CRC cells in a wound-healing assay. Profiling a panel of CRC cell lines for growth inhibitory effects showed that 20-223 has nanomolar potency across multiple CRC cell lines and was on an average >2-fold more potent than AT7519. Cell cycle analyses in CRC cells revealed that 20-223 phenocopied the effects associated with AT7519. Collectively, these findings suggest that 20-223 exerts anti-tumor effects against CRC by targeting CDK 2/5 and inducing cell cycle arrest. Our studies also indicate that 20-223 is a suitable lead compound for colorectal cancer therapy.

## INTRODUCTION

Colorectal cancer (CRC) continues to be a major health concern in the United States where it is currently the fourth most commonly diagnosed malignancy and the second leading cause of cancer related deaths [1]. Despite numerous attempts at developing promising therapies for CRC, few have successfully improved patient outcome.

Cyclin-dependent kinases (CDKs) have been extensively studied and characterized for their roles in cancer. There are 20 members of the CDK family, all of which have been linked to cancer. CDKs are often categorized into two major groups, those that contribute to tumorigenesis through cell cycle control and those that regulate transcription [2–4]. One peculiar member of the CDK family that does not regulate transcription and only recently has been shown to contribute to cell cycle progression, is CDK5. Uniquely, CDK5 is not activated in typical CDK fashion i.e., through binding of cyclins, but instead is activated by regulatory proteins p35 and p39 [5]. CDK5 is best known for its role in the central nervous system where it regulates development, axon elongation, synaptogenesis and neuronal migration. Recently, reports have identified CDK5 as a key player in non-neuronal functions including apoptosis, senescence, angiogenesis, insulin secretion, wound healing, and adhesion/migration [6]. These functions associated with CDK5 are believed to contribute to its role in tumorigenesis. CDK5 has been previously implicated in a number of cancers, including those of the pancreas [7, 8], thyroid [9, 10], prostate [11, 12], breast [13], lung [14], liver [15], and most recently as a tumor promoter in CRC [16].

CDKs have received a lot of attention as potential targets for cancer therapy. The traditional approach to targeting CDKs, which still remains popular, is through the use of ATP-competitive inhibitors that bind within the catalytic sites of CDKs and outcompete the binding of ATP. The earliest CDK inhibitors were pan-CDK inhibitors that often targeted most, if not all the members of the family. While they showed promise in targeting CDKs, they often required high doses which resulted in off-target effects and significant toxicity in preclinical animal trials [17]. To address these issues, substantial efforts have been made to improve upon the potency of CDK inhibitors. While CDK inhibitors are currently being used to treat a variety of malignancies, few are currently being tested in CRC [18].

ATP competitive inhibitors typically form hydrogen bonds with the residues in the hinge region of the kinase. Aminopyrazole is a privileged scaffold that forms a network of hydrogen bonds between 3 nitrogen atoms of the scaffold and the hinge region of the kinase [19, 20]. AT7519, a well-characterized pan-CDK inhibitor built on a 4-aminopyrazole core has shown promise in pre-clinical and clinical studies [21–23].

Herein, we describe our findings with a 3-aminopyrazole analog previously reported by Pfizer (CP668863 a.k.a 20-223), that was developed to treat neurodegenerative disorders [23]. Preliminary xenograft studies showed 20-223 reduced tumor growth and tumor weight *in vivo* indicating that 20-223 is a suitable lead compound for CRC therapy. We subjected 20-223 and AT7519 to a series of cell-free and cell-based assays to understand the mechanistic basis of the observed 20-223 anti-tumor effects. Docking studies suggested both 20-223 and AT7519 are ATP competitive inhibitors. The two aminopyrazole analogs were compared head-to-head in cell free kinase assays which demonstrated 20-223 was more potent than AT7519. Contrary to a previous report, we found 20-223 was equipotent against CDK2 and CDK5 compared to other members of the CDK family. Examination of downstream substrate phosphorylation showed 20-223 inhibited the kinase activity of CDK2 and CDK5. Migration studies utilizing a wound-healing assay showed that 20-223 decreased CRC cell migration. 20-223 was a nanomolar inhibitor of cell growth in a panel of CRC cell lines and was more potent than AT7519. Finally, 20-223 phenocopied cell cycle effects associated with AT7519. Together, our studies suggest 20-223 is a CDK 2/5 inhibitor, an effective anti-CRC agent and suitable lead for pre-clinical development.

## RESULTS

### TCGA analyses reveals CDK5 is upregulated in primary colorectal tumors as a result of increased copy number

With increasing evidence suggesting a role for CDK5 in a variety of malignancies, we turned to The Cancer Genome Atlas (TCGA – http://cancergenome.nih.gov/) database to gain insight into CDK5 expression in patient populations. We found the colorectal cancer cohort in TCGA online database consisted of 50 samples of normal mucosa and 347 primary colorectal tumor samples. The mRNA profiles of these samples were examined for CDK5 expression. As seen in Supplementary Figure 1A, CDK5 mRNA levels were significantly higher in primary tumor compared to normal colon. Additional analyses that compared normal tissue with corresponding primary tumor revealed that of the 31 patients examined, all but two showed a significant increase in CDK5 levels in primary tumors when compared to normal colon tissue (Supplementary Figure 1B). Next we examined the CDK5 copy number to determine whether increased CDK5 levels correspond to increased copy number. Of the 616 sequenced CRC samples, few exhibited homozygous deletion or heterozygous loss of CDK5 (0.3% and 1.9% respectively). Interestingly, 46.0% of individuals were diploid for CDK5 while 51.9% of individuals had a copy number gain for CDK5 (Supplementary Figure 1C). Additionally, we found that across all four groups, there is a significant linear trend. As copy number of CDK5 increases there is a corresponding increase in mRNA expression (Supplementary Figure 1D) thus suggesting that copy number is a contributing factor to increased mRNA expression that we observed in CRC. Next, we investigated whether CDK5 mutation could possibly be contributing to its activity in CRC so we examined the mutational frequency of CDK5 in all TCGA cancers. We found that CDK5 is rarely mutated across cancers and more importantly is not mutated in CRC (Supplementary Figure 1E). Collectively, this data suggests that CDK5 activity is a result of increased expression that results from an increase in copy number. Furthermore, it is the increase in CDK5 expression, not a mutation, which is likely responsible for its contributions to CRC. These data are consistent with a recent report implicating CDK5 as a tumor promoter in CRC and thus warrants the investigation into inhibition of CDK5 as a potential therapeutic option for CRC [16].

### 20-223 shows anti-tumor activity in human CRC xenograft tumors

**Figure 1:**
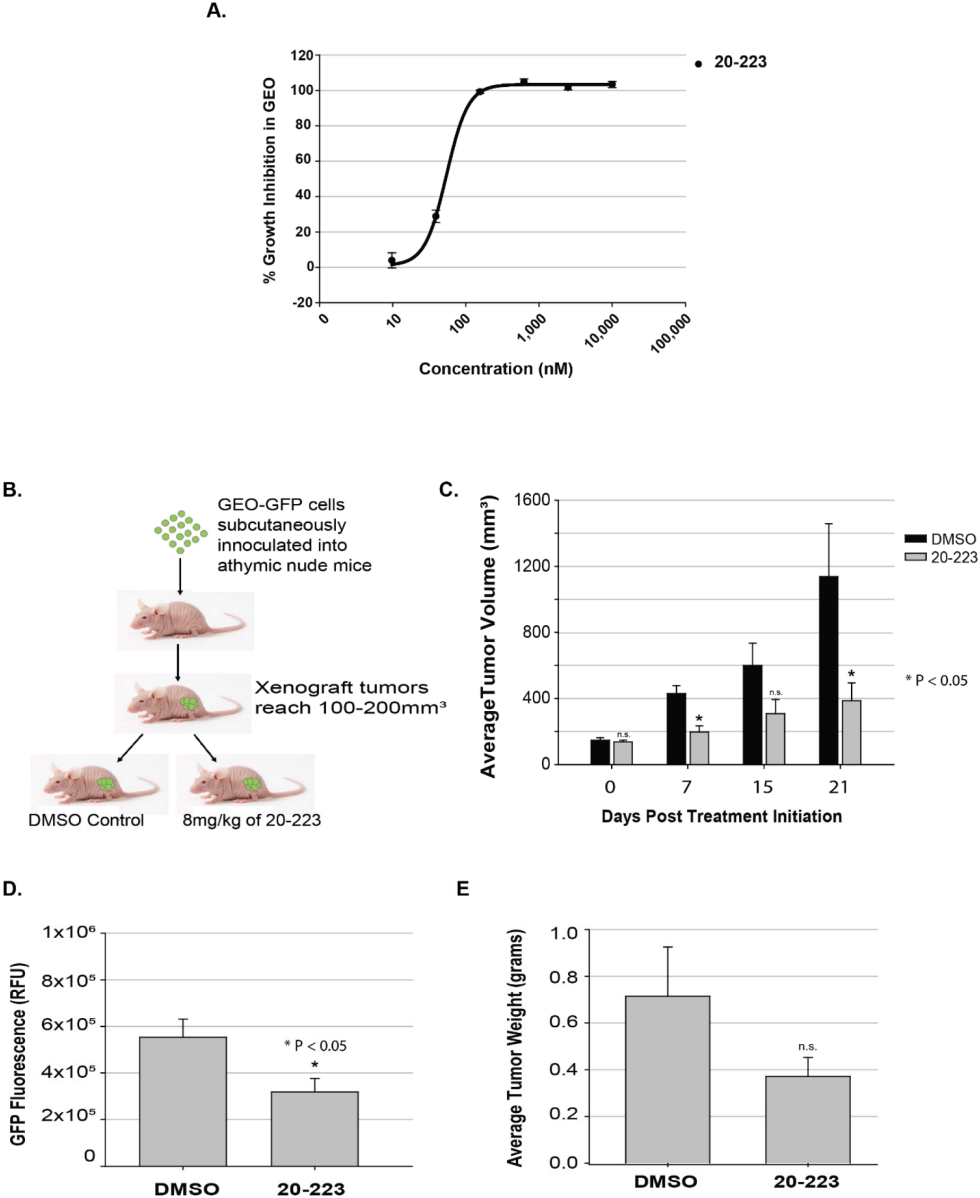
20-223 exhibits anti-tumor activity in a colorectal cancer xenograft model **(A)** Growth Inhibition of GEO cells after 72 hour treatment with 20-223. **(B)** Schematic representation of CRC xenograft model using GEO-GFP cells. **(C)** Average tumor volume comparison of DMSO and 20-223 treated tumors throughout the study. **(D)** Average GFP Flurorescence in DMSO and 20-223 treated tumors. **(E)** Average tumor weight of DMSO and 20-223 treated tumors.

CP668863, a substituted 3-aminopyrazole analog, was first reported by Pfizer as an ATP-competitive CDK5 inhibitor that was explored for the treatment of neurodegenerative disorders [24]. With increasing evidence that CDK5 activity contributes to CRC tumorigenesis, we synthesized CP668863 (a.k.a 20-223) to screen for its efficacy against CRC. We utilized our well established CRC xenograft model [25] to determine the effects of 20-223 *in vivo*. GEO cells were chosen because they form primary tumors 100% of the time and frequently exhibit metastatic spread (approximately 53%) in animal models [26]. As our xenograft model uses GEO cells, we performed an initial growth inhibition study to show efficacy of 20-223 in this cell line. We found 20-223 to have an IC_50_ value of 79nM in GEO cells (Figure 1A). We used a GEO cell line in which GFP is stably expressed, for our xenograft model. GEO-GFP cells were subcutaneously injected into the flank of athymic nude mice and allowed tumors to grow to ∼100 mm^3^. Animals with tumors were then randomly divided into two treatment groups (I) DMSO or (II) 8mg/kg 20-223. In preliminary PK studies, mice were dosed with 8mg/kg of 20-223. The plasma concentration was greater than 79nM for 24 hours as determined by LC-MS (Supplementary Figure 2A). Subcutaneous injections were given in the shoulder area of each mouse daily for the first week and every other day for the following two weeks (Figure 1B). The mice were weighed and tumor volumes were measured every other day. At the end of the three-week treatment period the mice were euthanized and the tumors excised, weighed and imaged (Supplementary Figure 2B).

Average changes in tumor volume for both treatment groups are summarized in Figure 1C. At the end of the first week of treatment, average tumor volume in the DMSO-treated group was approximately 2-fold greater than average tumor volume in the 20-223-treated group (∼429mm^3^ vs. ∼197mm^3^). The tumor volumes of DMSO-treated animals continued to grow rapidly, while the tumor progression in 20-223-treated animals was slower (Figure 1C). At the conclusion of the study, tumors from the DMSO treated mice were significantly larger (∼3-fold: 1138 mm^3^ vs. 386 mm^3^) than the tumors from the 20-223-treated mice. We also found a decrease in GFP fluorescence in 20-223-treated tumors compared to DMSO-treated tumors, which is consistent with the tumor volume trends (Figure 1D). The average tumor weights from DMSO-treated mice were also ∼2-fold greater than tumors from 20-223-treated mice (0.7g vs. 0.3g) (Figure 1E). Of note, 20-223 treated animals did not exhibit any overt signs of toxicity, as there was no change in animal weight or behavior.

To confirm inhibition of CDK5 *in vivo*, we performed western blot analyses on the tumor lysates with tumors from three representative animals from each treatment group. 20-223-treated tumors showed a decrease in the pFAK levels, a phosphorylation site specific to CDK5 [27], (Supplementary Figures 2C) suggesting inhibition of CDK5 *in vivo*. In summary, these studies suggest that 20-223 treatment results in anti-tumor activity in a CRC xenograft model.

### 20-223 is an ATP-competitive inhibitor

Our *in vivo* data suggests 20-223 may be a promising therapeutic agent for CRC, therefore we began to evaluate and characterize it in cell-free and cell-based studies. We started with docking studies that compared 20-223 to another known CDK inhibitor, AT7519, which is currently in clinical trials and shares the aminopyrazole core structure with 20-223 [21, 23].

**Figure 2:**
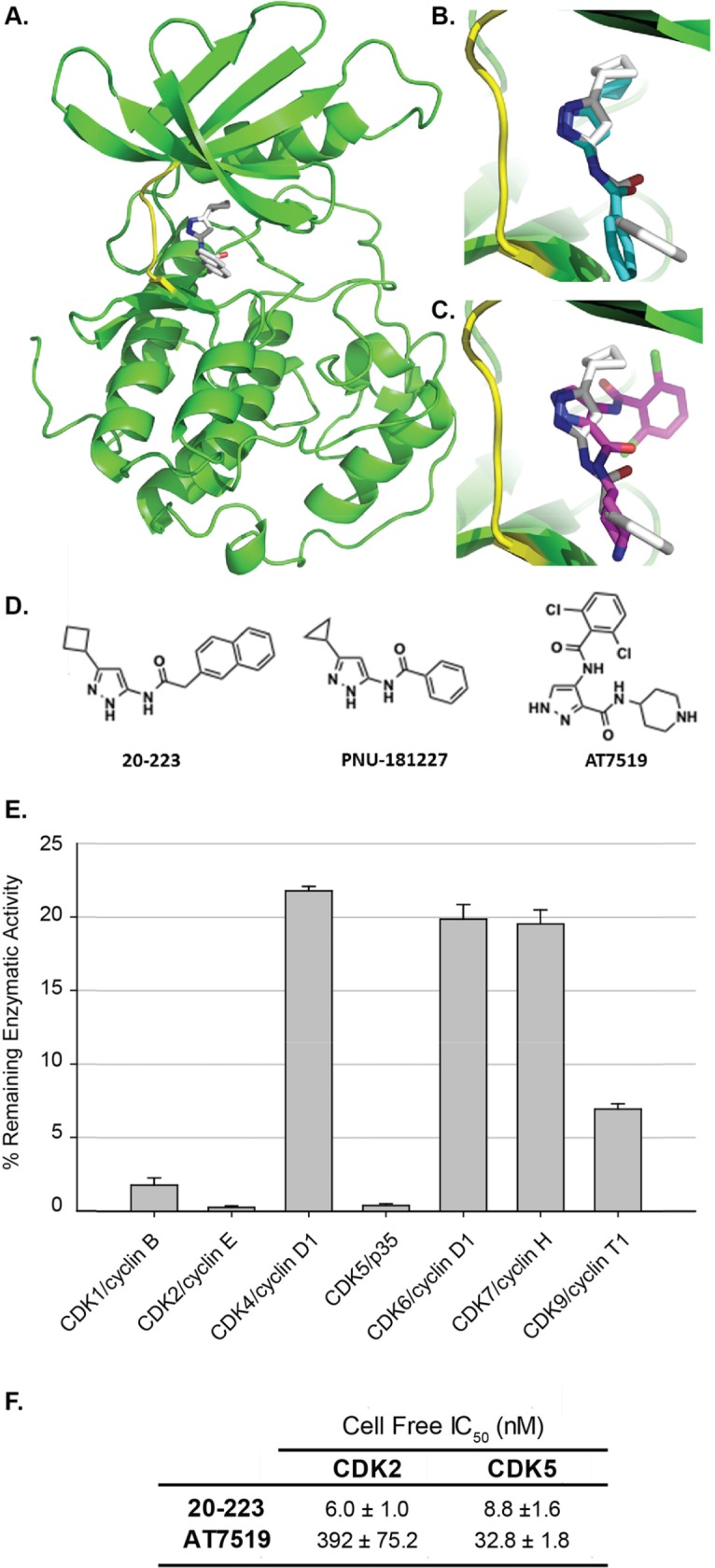
Structural and cell free analyses of 20-223 and AT7519 **(A)** Docking of 20-223 into CDK5 using AutoDock Vina software. **(B)** Overlay of 20-223 and PNU181227 in the hinge region of CDK5. **(C)** Overlay of 20-223 and AT7519 in the ATP binding pocket. **(D)** Chemical structures of 20-223, PNU-181227, and AT7519. **(E)** % of remaining enzymatic activity of a panel of CDKs after incubation with 0.1μM 20-223 and 30μM ATP. **(F)** IC_50_ values (nM) of CDK2 and CDK5 after incubation with 20-223 or AT7519 in cell free dose-escalation study.

X-ray crystallographic studies of reported aminopyrazole analogs and CDKs showed that they occupy the ATP binding site in the CDKs [18, 19]. Since there is no co-crystal structure of 20-223, we docked 20-223 into CDK5 using Autodock Vina to explore its binding mode. Our docking studies revealed that 20-223 indeed occupied the ATP binding site of CDK5 and the three nitrogen atoms of the 3-aminopyrazole core are involved in a donor-acceptor-donor hydrogen bond triad with Glu81 and Cys83 of the hinge region. The cyclobutyl ring occupied a narrow hydrophobic pocket formed by Phe80, Leu55 and Val64 and the naphthalene ring of 20-223 is directed towards the solvent-accessible region of the kinase (Figure 2A). Since CDK2 and CDK5 share sequence homology of ∼60% (5), we overlaid the co-crystal structure of an aminopyrazole analog PNU-181227-CDK2 with our docked 20-223-CDK5 and observed similar binding mode (Figure 2B). X-ray crystallographic studies demonstrated AT7519 to be an ATP-competitive CDK inhibitor [21]. Overlay of AT7519 complexed with CDK2 and docked 20-223-CDK5 showed similar mode of binding with similar hydrogen bonding interactions anchoring the molecules to the hinge region (Figure 2C). The chemical structures of all three of these compounds are compared in Figure 2D.

### Cell free kinase assays reveal 20-223 is a CDK 2/5 inhibitor

In order to determine the selectivity profile of 20-223 for various CDKs we conducted a single dose kinase screen with a panel of CDKs. Members of the CDK family bound to their respective activators were incubated with 0.1μM of 20-223 and 30μM ATP. The percentage remaining enzymatic activity was determined for each of the examined CDKs after inhibition by 20-223 (Figure 2E). Incubation with 20-223 markedly inhibited the enzymatic activity CDK2 and CDK5 with only 0.26% and 0.39% enzymatic activity remaining. 20-223 was less effective against the enzymatic activity of CDK1, CDK4, CDK6, CDK7, and CDK9. These results show that 20-223 is most effective against CDK2 and CDK5 in a cell-free system. To determine IC_50_ values of 20-223 against CDK2/5 we performed a dose-response study. CDK2/CyclinE and CDK5/p35 were incubated with 20-223 at various concentrations and IC_50_ values of 6.0nM for CDK2 and 8.8nM for CDK5 were derived from curve fitting the data (Figure 2F). Similar studies were also carried out with AT7519 and IC_50_ values of 392nM for CDK2 and 32.8nM for CDK5 were obtained. Results from the dose-response study show that 20-223 is equipotent against CDK2 and CDK5 in a cell-free system and is more potent than comparable CDK inhibitor, AT7519.

### CDK2 and CDK5 expression and phosphorylation activity in a panel of human CRC cell lines

**Figure 3:**
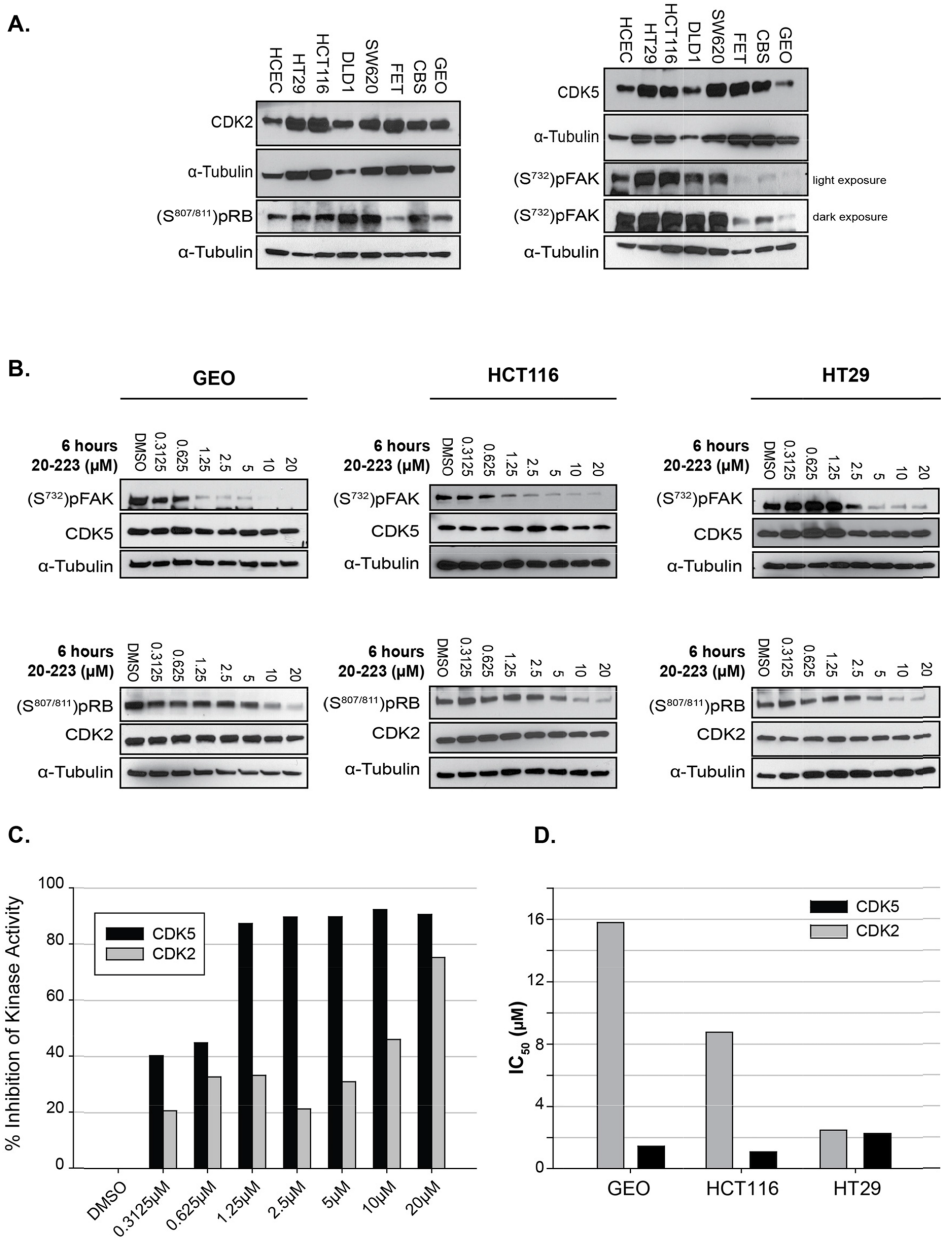
20-223 inhibits the kinase activity of CDK5 and CDK2 *in vitro* **(A)** Baseline expression of CDK2 and pRB (S807/811) (left), CDK5 and pFAK (S732) (right), in untreated CRC cells. **(B)** Representative western blots of target substrate pRB and pFAK phosphorylation levels in GEO (left), HCT116 (middle) and HT29 (right) cell lines after 6 hour incubation with 20-223. **(C)** Representative quantification of % inhibition of CDK2 and CDK5 kinase activity (based on substrate phosphorylation levels) in GEO cells found in Figure 3B. **(D)** Cell-based IC_50_ values generated from phosphorylation levels in Figure3B of CDK2 and CDK5 in three CRC cell lines.

Having determined that 20-223 targets CDK2 and CDK5, we next examined the basal levels of these kinases in a cohort of colorectal cancer cell lines which includes seven CRC cell lines and one normal human colon epithelial cell line (HCEC). All the cell lines expressed CDK2 and CDK5, albeit at different levels. HCEC cells also expressed CDK2 and CDK5 but at much lower levels than many of the CRC cell lines (Figure 3A). This observation is consistent with the TCGA data.

As a measure of CDK2 and CDK5 activity we examined basal phosphorylation levels of substrates specific to CDK2 and CDK5. Phosphorylation levels of RB (S807/811) were used as a read-out for CDK2 kinase activity. While CDK4 has also been shown to phosphorylate RB at S807/811 [28, 29], our kinase profile screen showed 20-223 targets CDK2/5 more effectively than CDK4/6. Phosphorylation levels of FAK (S732) were used as a read out for CDK5 kinase activity [27] [28]. We observed differential phosphorylation of RB (S807/811) and FAK (S732) indicating both CDK2 and CDK5 are active in each of the cell lines (Figure 3A).

### 20-223 disrupts CDK2 and CDK5 kinase activity in cell-based studies

Since 20-223 was shown to most potently inhibit CDK2 and CDK5 in a cell-free system, we next explored the ability of 20-223 to target CDK2 and CDK5 in a cellular setting. To characterize the effects of 20-223 on substrate phosphorylation, three CRC cell lines were chosen: GEO, HCT116 and HT29. CRC cells were incubated with DMSO or various concentrations (20μM – 2-fold dilutions, 7 doses) of 20-223 for 6 hours prior to western blot analyses. In the dose response study, 20-223 did not affect the total levels of CDK2 or CDK5 (Figure 3B), nor did it affect the levels of total FAK or total RB (Supplementary Figure 3). As expected, 20-223 induced a dose-dependent decrease in pRB (S807/811) and pFAK (S732) levels in each of the three CRC cell lines (Figure 3B). Quantification of phosphorylated RB and FAK levels was performed to reveal the effect of the inhibitor on CDK2 and CDK5 kinase activity, respectively. As the concentration of 20-223 increased, there was a corresponding increase in % kinase inhibition for CDK2 and CDK5 (Figure 3C). This pattern was consistent for each of the three cell lines. Quantification was also used to assess the fold selectivity of 20-223 in each of the three cell lines. Figure 3D summarizes the cell-based IC_50_ values for each cell line. 20-223 was ∼10 fold more selective for CDK5 over CDK2 in GEO cells (1.44μM vs 15.79μM) and ∼8-fold more selective for CDK5 over CDK2 in HCT116 cells (1.08μM vs 8.76μM), However, in HT29 cells, 20-223 was equally potent against CDK5 and CDK2 (2.45μM vs. 2.25μM). While the generated IC_50_ values are based on a qualitative observation, these results demonstrated that 20-223 effectively blocks the kinase activity of CDK2 and CDK5 in multiple CRC cell lines.

### 20-223 reduces migration of CRC cells

**Figure 4:**
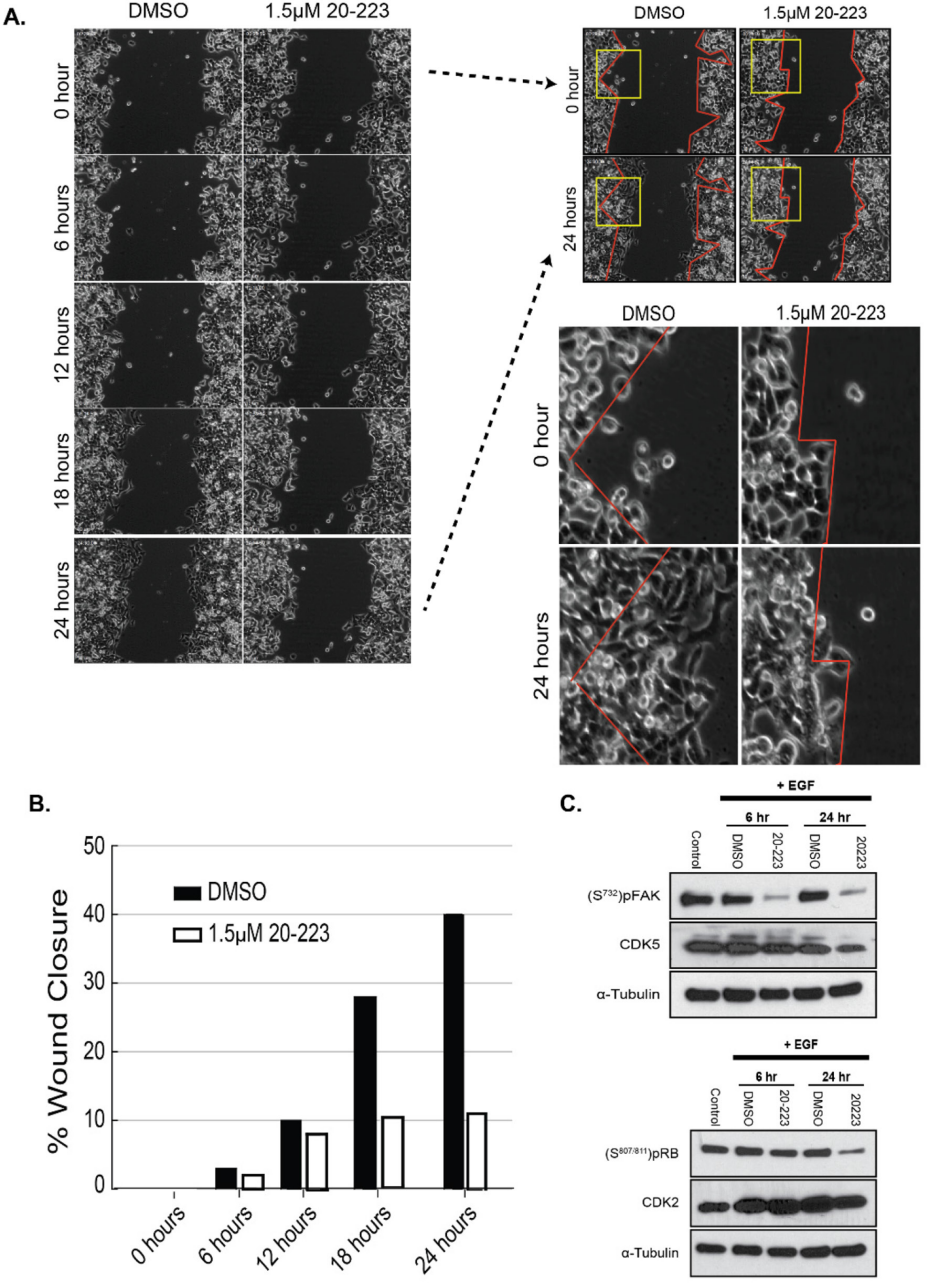
20-223 disrupts migration of CRC cells **(A)** Wound gap images taken during the 24 hour incubation of HCT116 cells with DMSO or 1.5μM 20-223. 0 and 24 hour images were further evaluated by outlining the wound area (red lines) and zooming in on the wound boundaries (yellow box). **(B)** Quantification of % wound closure after treatment of HCT116 cells with DMSO of 1.5μM 20-223. **(C)** Western blot analyses at 6 and 24 hours after stimulation with EGF and treatment with either DMSO or 1.5μM 20-223.

Since 20-223 effectively inhibits CDK2 and CDK5, both of which have previously been shown to regulate cell motility [27, 30], we next examined its ability to disrupt CRC cell migration. Wound-healing scratch assays are routinely used to assess the effect of small molecule inhibitors on the ability of cells to migrate [31]. EGF-stimulated wound healing has previously been shown to enhance migration of cells; therefore, we used this ligand to stimulate CRC cells for migration [32]. We checked protein levels of CDK2/5 and their substrates after EGF stimulation (100ng/mL) to ensure that treatment with EGF would not affect their basal levels or activity. Upon treatment with EGF, no changes in the levels of CDK2/5 or pRB/pFAK were observed, indicating that EGF is not affecting the expression or activity of these kinases (Supplementary Figure 4A). HCT116 cells were used to model cell migration because they have been used previously in wound-healing scratch assays [35]. To assess the ability of 20-223 to inhibit migration, HCT116 cells were stimulated with EGF and treated with DMSO or 1.5μM of 20-223. Live cell imaging was utilized to monitor cell motility through the 24 hour incubation period at 15 min intervals (Supplementary Figure 4B & 4C). Still images and zoomed in regions of the images emphasize the ability of 20-223 to inhibit cell migration (Figure 4A). HCT116 cells treated with DMSO had greater ability to migrate into the open wound areas compared to cells treated with 20-223. While cells treated with DMSO were able to close approximately 40% of the wound area, cells treated with 20-223 only closed approximately 10% of the wound (Figure 4B). To confirm that the reduced migration was a result of CDK2/5 inhibition, corresponding western blots were performed under the same conditions as the migration experiment and pFAK and pRB levels were determined after treatment with EGF and 1.5μM of 20-223. Although treatment with 20-223 effectively reduced the phosphorylation levels of both FAK (S732) and RB (S807/811) (Figure 4C), the effects were more pronounced on the FAK phosphorylation over RB phosphorylation. Collectively, these results suggest that inhibition of CDK2/5 by 20-223 disrupts CRC cell migration.

### 20-223 reduces cell growth in a panel of human CRC cell lines

**Figure 5:**
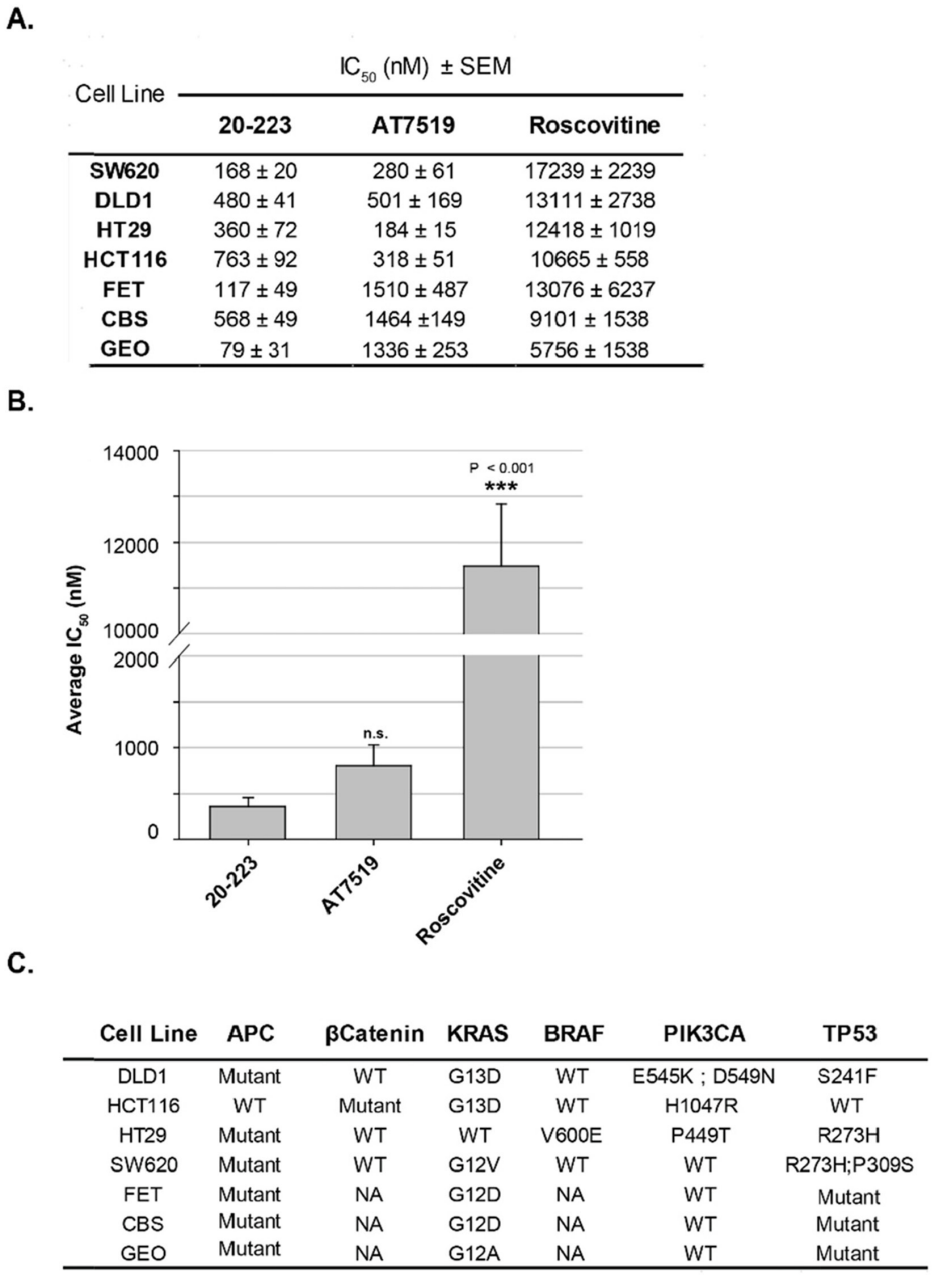
20-223 potently inhibits cell growth in a panel of CRC cell lines **(A)** IC_50_ values from growth inhibition studies after CRC cells were treated with 20-223, AT7519 or Roscovitine for 72 hours. **(B)** Average IC_50_ values across all seven CRC cell lines after treatment with 20-223, AT7519, or Roscovitine (P < 0.001). **(C)** Panel containing the seven CRC cell lines used in this study and mutational status of important regulatory genes.

Since 20-223 effectively targets CDK2 and CDK5, we next examined its effect on cell growth. We subjected a panel of CRC cell lines to treatment with three CDK inhibitors (20-223, AT7519 or Roscovitine). Roscovitine, which contains a purine core, was one of the first CDK inhibitors to enter clinical trials. CRC cells were treated with 20-223, AT7519 and Roscovitine at four-fold dilutions starting at 10μM (20-223 and AT7519) or 100μM (Roscovitine). Among the three inhibitors, 20-223 had lower IC_50_ values when compared to the clinically used CDK inhibitors, AT7519 and Roscovitine (Figure 5A). Among the CRC cell lines, SW620, GEO and FET cells were the most sensitive to 20-223, whereas HCT116 and HT29 were more responsive to AT7519 treatment as evident by lower IC_50_ values. It is important to note that a ∼10-fold higher dose of Roscovitine was required to observe similar growth inhibitory effects. Average IC_50_ values were calculated across cell lines to determine the overall efficacy for each compound (Figure 5B). 20-223 had an overall average IC_50_ value of 362nM across seven cell lines, while AT7519 and Roscovitine had overall average IC_50_ values of 799nM and 11481nM respectively, thus suggesting 20-223 is a more potent inhibitor of cell growth compared to the clinical compounds. CRC mutational profiles [33–39] (Figure 5C) were examined to determine if the presence of any particular mutations made any cell line more or less responsive to treatment with 20-223. We did not find any obvious correlation between IC_50_ values and the mutational profile. Based on these findings, we conclude that 20-223 is a sub-μM inhibitor of CRC cell growth. Specifically, these data show that 20-223 is ∼2.2 fold and ∼31.7 fold more potent than AT7519 and Roscovitine, respectively. Therefore, 20-223 is comparable or marginally better than the CDK inhibitors advanced to the clinics.

### Reduced CRC cell growth and tumor growth induced by 20-223 is probably not due to the induction of apoptosis

To determine if induction of apoptosis was responsible for potent CRC cell growth inhibition, we examined the effect of 20-223 on Poly(ADP-ribose)polymerase (PARP) cleavage. PARP cleavage is one of the hallmarks of cell death and is widely used as a read-out of apoptosis in cancer research. To determine the effect of 20-223 on cell death, we evaluated the panel of CRC cells treated with 20-223 for PARP cleavage. CRC cell lines treated with 20-223 for 24 hours all exhibited PARP cleavage (Supplementary Figure 5A). Next, we performed a dose-response and a time course study in GEO cells and found that μM concentrations of 20-223 and long exposure were required to induce apoptosis (Supplementary Figure 5B and 5C). This suggests that the reduced tumor growth observed in the mouse model is not due to induction of apoptosis.

### Reduced CRC cell growth and tumor growth in mice is probably due to the induction of cell cycle arrest by 20-223

**Figure 6:**
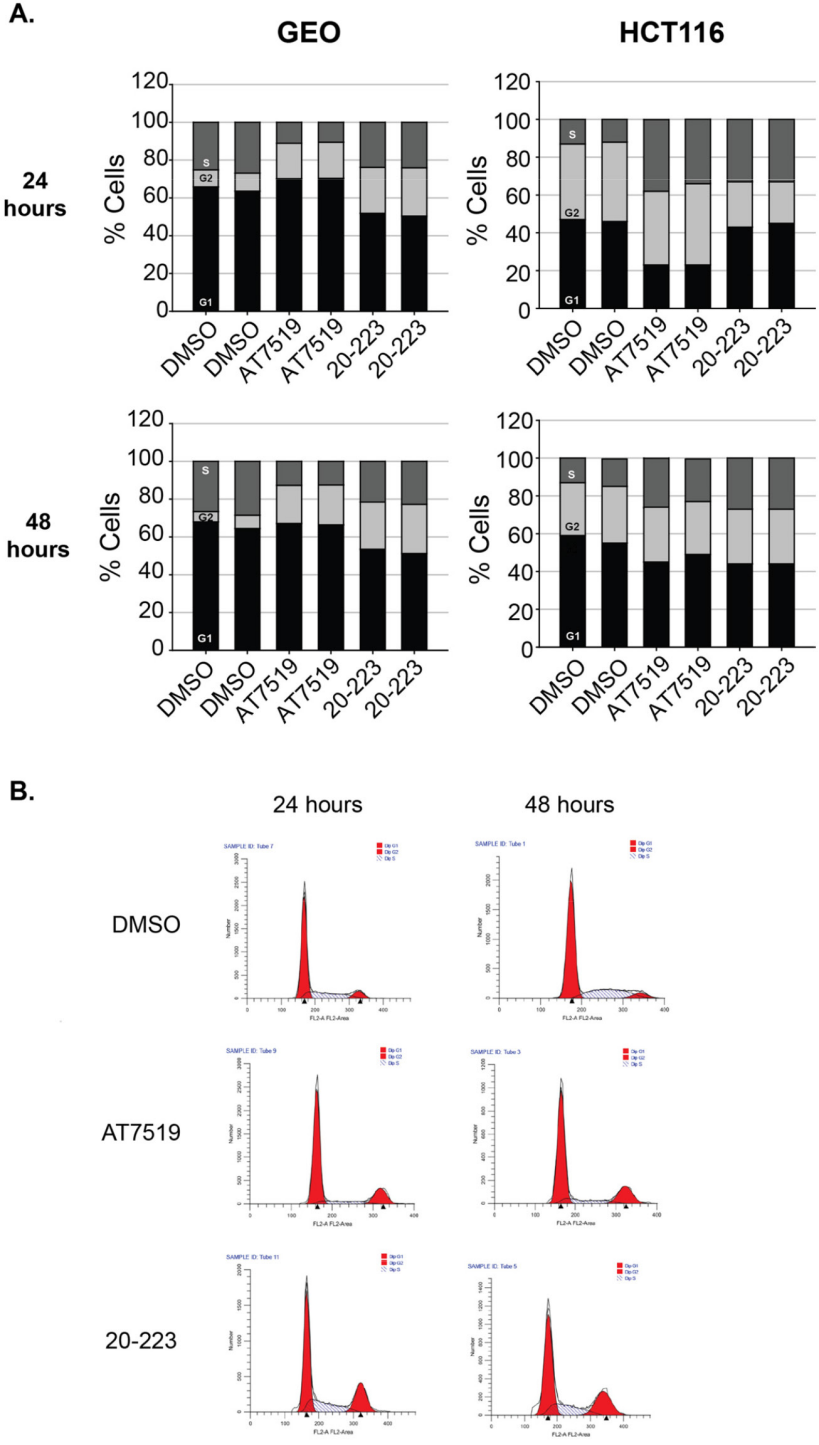
20-223 treatment in CRC cells results in cell cycle arrest *in vitro* **(A)** % of cells in each phase of the cell cycle after treatment with DMSO, AT7519, or 20-223 for 24 (top) and 48 (bottom) hours in GEO (left) and HCT116 (right) cells. **(B)** Traces representative of cell cycle analysis in GEO cells after treatment with DMSO, AT7519, or 20-223 after 24 (left) or 48 (right) hours.

The CDK family has been extensively studied for its regulation of all phases of the cell cycle. This kinase family is essential for normal cells to proliferate and divide. CDK2 has been shown numerous times to be required for progression from G1 and S phase of the cell cycle [40]. The role of CDK5 in cell cycle is less understood however, recent reports suggest it regulates the cell cycle through mitotic control and dysregulation of cell cycle inhibitors, p21^CIP1^ and p27 [41–43]. Having shown that 20-223 effectively targets CDK2 and CDK5 and also decreases cell growth, we sought out to understand how it may alter cell cycle progression. GEO and HCT116 cells were treated with either DMSO, AT7519 or 20-223 for 24 and 48 hours and then analyzed for DNA content by flow cytometry. The results from the above experiment are summarized in Figure 6A. 20-223 and AT7519 both effectively arrested the CRC cells in either the G2 or S phase of the cell cycle. GEO cells treated with either 20-223 or AT7519 arrested in G2 phase of the cell cycle (Figure 6B). This is consistent with the previous findings that CDK2 regulates the G2/M checkpoint in the absence of functional p53 [44]. Profiling the GEO cell line indicates that GEO cells carry a p53 mutation, therefore the G2/M arrest seen in GEO cells may be due to CDK2 inhibition. Alternatively, the G2 arrest could also be attributed to CDK1 inhibition as it was the third CDK inhibited in our profiling. HCT116 cells treated with either 20-223 or AT7519 resulted in S-phase arrest at the 24 and 48 hour time point. Figure 6B shows representative traces from the cell cycle analyses. The data clearly shows that 20-223 mimics the effects observed with AT7519. This data suggests that the observed CRC cell growth inhibition and the tumor growth in mice induced by 20-223 were due to cell cycle arrest.

## DISCUSSION

There is a need for targeted agents with defined mechanism of action for CRC therapy. Recent studies have validated CDK5 as a tumor promoter and designated it as a therapeutic target for CRC therapy. In the present study, we evaluated 20-223 (CP668863), a previously identified CDK5 inhibitor, for its potential as an anti-CRC agent. In a proof of concept study, we used an established CRC xenograft model to show that 20-223 effectively slowed tumor progression. Tumors in mice treated with 20-223 had reduced tumor volumes and tumor weights compared to vehicle-treated mice. Moreover, we observed lower levels of phosphorylated FAK, a well-characterized target of CDK5, in 20-223 treated tumors as compared to vehicle-treated tumors. These results are consistent with the studies reported with the neurodegenerative model [24].

Having successfully shown that 20-223 slows tumor progression *in vivo*, we followed up with characterization of the mechanistic basis for the observed anti-CRC effects in cell-free and cell-bases studies. For these studies, we used AT7519 and Roscovitine, both CDK inhibitors previously explored as anti-cancer CDK inhibitors in clinical trials. AT7519 and 20-223 share the same core structure, which makes it an optimal compound to benchmark the potency of 20-223.

We performed a series of studies to gain insight into the mechanism associated with the anti-tumorigenic properties elicited by 20-223. The aminopyrazole core found in CDK inhibitors has proven successful due to the flat heterocyclic core and a series of hydrogen bond donors and acceptors. The positioning of nitrogen atoms in the aminopyrazole core enables them to compete with ATP. A hydrogen bond donor acceptor donor triad within the aminopyrazole core targets the hinge region residues of the kinase and blocks the binding of ATP [45]. Docking studies suggested that aminopyrazole analogs 20-223 and AT7519 interact with Glu81 and Cys83 within the hinge region of CDK5. Profiling 20-223 against a panel of CDKs revealed that it most potently inhibits CDK2 and CDK5 over other CDKs. Importantly, 20-223 is more potent than the clinically used 4-aminopyrazole analog AT7519 in cell-free kinase assays.

Cell-based studies corroborated cell-free kinase assays as 20-223 effectively disrupted the kinase activity of CDK2 and CDK5 in CRC cells. In two of the three cell lines profiles 20-223 was selective for CDK5 over CDK2. The observed differential sensitivity/selectivity associated with 20-223 in three different CRC cell lines suggests that the functional misregulation of CDKs is probably not the same across the cell lines. The cell free and cell-based IC_50_ values were approximately two-orders of magnitude apart. This loss of potency when going from a cell free to cell-based activity assay is commonly observed in drug discovery programs. For example, Palbociclib the recently approved CDK4/6 inhibitor has single digit nM potency in cell free assays and has a single digit μM potency in cell based assays [46]. One possible explanation for this observed difference is the emerging view that kinases are part of larger protein complexes and evaluating selectivity in cell-free conditions does not always reflect the effects observed in the cellular context [47].

Since 20-223 showed ∼8-fold selectivity for CDK5 over CDK2 in HCT116 cells we evaluated its efficacy in inhibiting migration of HCT116 cells in a wound-healing scratch assay. 20-223 treated HCT116 cells showed reduced cell migration when compared to vehicle treated HCT116 cells. This is consistent with reported literature that shows that CDK5 plays an important role in regulating the migration of cells by phosphorylation of Ser732 on FAK [27]. Studies with a CDK2 inhibitor also showed it blocked EMT and subsequent cell migration, however in that study the effect of the inhibitor on CDK5 was not determined [30].

As CDK2/5 have been known to drive proliferation of cancer cells, we investigated the effect of 20-223 on cell growth in a larger panel of CRC cell lines. Among these CRC cell lines, 20-223 not only proved to be a nanomolar inhibitor of cell growth across the panel, but it was also more potent when compared to AT7519 and Roscovitine. These results suggest that 20-223 is comparable or in some cases more potent than the benchmarked clinical aminopyrazole analog AT7519.

Broadly the cause of CRC cell growth inhibition or tumor growth inhibition could be either due to induction of apoptosis or due cell growth arrest. A dose and time dependent study with 20-223 revealed that CRC cells required μM concentrations to induce PARP cleavage a hallmark for the induction of apoptosis. However at high nM to low μM concentrations of AT7519 or 20-223 we observed cell cycle arrest. Together our data shows that 20-223 phenocopies the cell cycle effects of AT7519 in CRC cell lines. The observed CRC growth inhibition can be largely attributed to inhibition of proliferation and to a lesser extent on the induction of apoptosis.

In summary, our study argues for the continued preclinical development of 20-223 for CRC therapy. Collectively, our results reveal that 20-223 exhibits anti-cancer properties in a CRC mouse model. Mechanism studies indicate that it inhibits CDK2/5 both *in vitro* and in CRC cell lines. Migration of CRC cells was inhibited by 20-223, which targeted CDK5 and as a consequence inhibited Ser732 phosphorylation a key event in the migration of cells. 20-223 inhibits proliferation of CRC cell lines by inducing cell cycle arrest. A recent review article outlined in detail the contributions of CDK5 to many types of cancer, supporting its potential as a novel target for cancer therapy across many tumor types [48]. While we demonstrated 20-223 is not selective for CDK5, it does indeed inhibit CDK5 *in vitro* and *in vivo*. 20-223 had comparable or in several assays better potency than the clinically used aminopyrazole CDK inhibitor AT7519, which is a good benchmark for advancing a compound through development. In order to explore this core for improved selectivity, structure activity relationship studies are currently underway in our lab and will be reported in due course.

## MATERIALS AND METHODS

### Chemical inhibitors

20-223 was originally designed and synthesized by Pfizer (CP668863) and resynthesized in our laboratory (20-223) (Supplementary Figure 6). Chemical structure was confirmed using proton and carbon NMR (Supplementary Figure 7) and HRMS (Supplementary Figure 8A). The purity of the compound was analyzed by analytical HPLC (Supplementary Figure 8B). AT7519 (SelleckChem S1524) and Roscovitine (Apex BioTech #A1723) were used in head-to-head comparison studies with 20-223. All three inhibitors were dissolved in 100% DMSO to a final stock concentration of 10mM.

### CRC cell lines and reagents

Cell lines used in this study are colorectal cancer (CRC) cell lines. FET, CBS, and GEO cells were cultured in serum free medium (McCoy's 5A medium with sodium bicarbonate, L-serine, asparagine, sodium pyruvate, MEM vitamins, growth factors (4μg/mL transferrin, 20μg/mL insulin, and 10ng/mL EGF), and 1x Penicillin-Streptomycin. SW620, DLD1, and HT29 cells were cultured in DMEM high glucose medium (HyClone #SH30022.01) supplemented with 10% FBS (Gibco by Life Technologies #26140-079) and 1x Penicillin-Streptomycin (HyClone # SV30010). HCT116 cells were cultured in RPMI-1640 Medium (HyClone #SH30027.01) supplemented with 10% FBS and 1x Penicillin-Streptomycin. All CRC cell lines were cultured in 5% CO_2_ at 37°C. Cell lines were validated by STR profiling at the University of Nebraska Medical Center Human DNA Identification Laboratory (Supplementary Figure 9A). Cell lines exceeding an 80% match with the online ATCC database were considered valid [49, 50].

### Human colon epithelial cell line

Immortalized non-transformed human colonic epithelial cell lines (HCEC) were a gift from J. Shay (UT Southwestern)[51]. HCECs were grown in medium composed of 4 parts DMEM to 1 part media 199 (Sigma-Aldrich) with 2% cosmic calf serum (GE Healthcare), 25 ng/mL EGF, 1 μg/mL hydrocortisone, 10 μg/mL insulin, 2 μg/mL transferrin, 5 nM sodium selenite, and 50 μg/mL gentamycin sulfate. HCECs were grown in a hypoxia chamber with 2% O_2_ and 5% CO_2_ at 37°C.

### Xenograft studies

All animal studies were carried out following approval of the Institutional Animal Care and Use Committee. This xenograft study has been used previously in our lab [27]. Briefly, GEO-GFP cells (7×10^6^) were subcutaneously injected into the flank of athymic nude mice. Xenograft tumors were allowed to grow until reaching a volume of 100-200mm^3^ at which point they were separated into two treatment groups: DMSO vehicle control or 8mg/kg 20-223. Each group contained 7 animals (n=7). Drug or vehicle injections were given subcutaneously daily for the first week and every other day for two more weeks for a total of 14 injections. Throughout the study, animal weight and tumor volume were measured. Tumor volume was measured with calipers and calculated using the l^2^ × h × π/6 equation. The study was concluded when control tumors reached maximum size according to facility guidelines. Mice were euthanized and then full body and excised tumor images were taken using Near-IR enhanced Macro Imaging System Plus Cooled with the LT-99D2 with the Dual Tool excitation upgrade. Tumor samples were preserved in liquid nitrogen prior to western blot analyses (see below for Western Blot protocol).

### The cancer genome atlas (TCGA) analyses

TCGA provisional data was retrieved from cBioPortal on January 19, 2017. CRC sample type and mRNA expression (RNA-seq) was downloaded from UCSC Xena (https://genome-cancer.soe.ucsc.edu/proj/site/xena/heatmap/). All provisional cancer datasets were analyzed for CDK5 mutation. The genomic profile of CDK5 was further analyzed in the CRC (Colorectal Adenocarcinoma - TCGA Provisional) dataset for putative somatic copy-number alterations from GISTIC, using Onco Query Language (OQL), and mRNA expression (RNA-seq). GISTIC predicts copy number alterations according to sample specific thresholds generated by comparing chromosomal segments with median chromosomal arm copy numbers. All parameters were set at default.

### Cell-free system analyses

Kinase profiling with 20-223 was carried out with a panel of CDKs (CDK1, CDK2, CDK4, CDK5, CDK6, CDK7, and CDK9) at a single dose (0.1μM) with 30μM ATP in duplicates. The enzymatic activity was determined for each of the CDKs and follow-up dose response studies were carried out with CDK2 and CDK5. A 10-point dose response starting at 5 μM of 20-223 or AT7519 with 3-fold dilution was carried out. IC_50_ values were generated through fitting the dose response curves.

### Western blot analyses

Cells were lysed using a buffer containing 50mM Tris, 100mM NaCl, 1% NP-40, 2mM EDTA, 20%SDS combined with 20xPPI (Na_3_VO_4_, NAF, β-glycerophosphate), and 1 mmol/L PMSF. Samples were kept on ice and vortexed prior to centrifugation at 4°C. Supernatant was collected and protein was quantified using BCA Protein Assay (Pierce # 23225). 40μg of protein were run on 4-15% gradient gels (BioRad) in 1x TRIS-Glycine SDS (Research Products International Corporation #T32080) at 120V for ∼90 minutes and separated by SDS-PAGE electrophoresis prior to being transferred to a PVDF membrane using a Semi-dry transfer (ThermoScienctific, #35035) at 18V for 35 minutes. The membrane was blocked in 5% milk in 1X Tris Buffered Saline with 0.1%Tween (1xTBST) for 1 hour at room temperature while gently rocking. Primary antibodies (Supplementary Figure 9B) were incubated in 5% milk in 1x TBST and rocked overnight at 4°C. Appropriate HRP-conjugated secondary antibodies were incubated in 5% milk in 1xTBST and rocked for 1 hour at room temperature. Protein expression was detected using ECL Prime (GE Healthcare #RPN2236). Kinase activity was measured by changes in substrate phosphorylation. Quantification of phosphorylation levels representative of the western blots shown were generated using ImageJ. Blots were performed in triplicate (n=3).

### Wound healing migration

HCT116 cells were plated at 1.25×10^6^ cells in 2mL medium in a 6-well plate and allowed to adhere overnight and reach 90% confluency. Cells were scratched using a sterile 10μL pipette tip down the middle of the well to create a “wound”. Scratched cells were washed gently with PBS before being stimulated with a final concentration of 100ng/mL of EGF (Invitrogen # PHG0311L) and immediately treated with either 1.5μM 20-223 or DMSO control. Directly after the start of treatment, cells were taken to the live cell imaging facility where they were imaged every 15 minutes over a 36 hour time course (only the first 24 hours were considered for migration purposes). Migration assays were performed in triplicate (n=3).

### Cell viability

CRC cells were plated at 4000 cells/well in a 96 well plate. Cells were treated with 20-223, AT7519 or Roscovitine at 4-fold dilutions starting at 10μM (20-223 and AT7519) or 100μM (Roscovitine) and incubated at 37°C for 72 hours. The ability of these compounds to inhibit cell growth was assessed using the dye PrestoBlue. Following a 15 minute incubation with PrestoBlue reagent (Invitrogen #A13262), fluorescence was measured at 560nM excitation and 590nM emission using SpectraMax M5*^e^*. Growth Inhibition was calculated using 100-[100^*^(Sample – T0)/(T100-T0)] equation, where T0 is the control reading immediately following treatment and T100 is the control reading at the end of a 72 hour incubation. Each assay was performed in triplicate (n=3).

### DNA-cell cycle analyses

CRC cells were plated at 1×10^6^ cells in a 10cm plate and allowed to adhere overnight. Cells were starved for 24 hours prior to treatments at 2x the growth inhibition IC_50_ values with 20-223, AT7519 or DMSO and were incubated for 24 and 48 hours prior to cell cycle analyses. 1×10^6^ cells were collected and pelleted by centrifugation at 2000 rpm for 1 minute at 4°C. Supernatant was decanted and pellets were resuspended in 1mL of 70% Ethanol and incubated at 4°C for 1 hour. Samples were centrifuged at 2000 rpm for 1 minute at 4°C and ethanol was removed. Pellets were washed 1x with 1mL of 1xPBS then centrifuged. PBS was removed and samples were resuspended in 1mL of Telford Reagent (115uM EDTA, 27μg/mL RNAseA, 50μg/mL Propidium Iodide, 0.1% Triton X-100, made in 1xPBS) and incubated at 4°C for 1 hr. Cells were analyzed for DNA content by flow cytometry. % of cells in the G1, G2, and S phases were determined for each treatment. (n=2).

### Statistical analyses

Graphs and figures were generated using SigmaPlot 11.0 and Graphpad Prism statistical software (GraphPad Software, Inc). Student's t-test was used to compare differences between means between two groups. One-way analyses of variance (ANOVA) with a post-test for linear trends was used to compare two or more groups. For all analyses, significance was inferred at P < 0.05 and P values were two-sided.

## SUPPLEMENTARY MATERIALS FIGURES


