# Correction: Characterization of CDK(5) inhibitor, 20-223 (aka CP668863) for colorectal cancer therapy

**DOI:** 10.18632/oncotarget.27403

**Published:** 2020-06-23

**Authors:** Caroline M. Robb, Smit Kour, Jacob I. Contreras, Ekta Agarwal, Carter J. Barger, Sandeep Rana, Yogesh Sonawane, Beth K. Neilsen, Margaret Taylor, Smitha Kizhake, Rhishikesh N. Thakare, Sanjib Chowdhury, Jing Wang, Jennifer D. Black, Michael A. Hollingsworth, Michael G. Brattain, Amarnath Natarajan

**Affiliations:** ^1^ Eppley Institute for Research in Cancer, University of Nebraska Medical Center, 985950 Nebraska Medical Center, Omaha, Nebraska 68198-5950, USA; ^2^ Department of Pharmaceutical Sciences, University of Nebraska Medical Center, Omaha, Nebraska 68198-5950, USA; ^3^ Section of Gastroenterology, Department of Medicine, Boston University Medical Center, Boston, Massachusetts 02118, USA; ^4^ Fred and Pamela Buffett Cancer Center, University of Nebraska Medical Center, Omaha, Nebraska 68198-5950, USA; ^*^ Deceased

**This article has been corrected:** The pRb blots in HCT116 and HT29 in Figure 3B were inadvertently duplicated during the assembly of the figure. The corrected Figure 3 is shown below. The authors declare that these corrections do not change the results or the conclusions of this paper.

Original article: Oncotarget. 2018; 9:5216–5232. 5216-5232. https://doi.org/10.18632/oncotarget.23749

**Figure 3:**
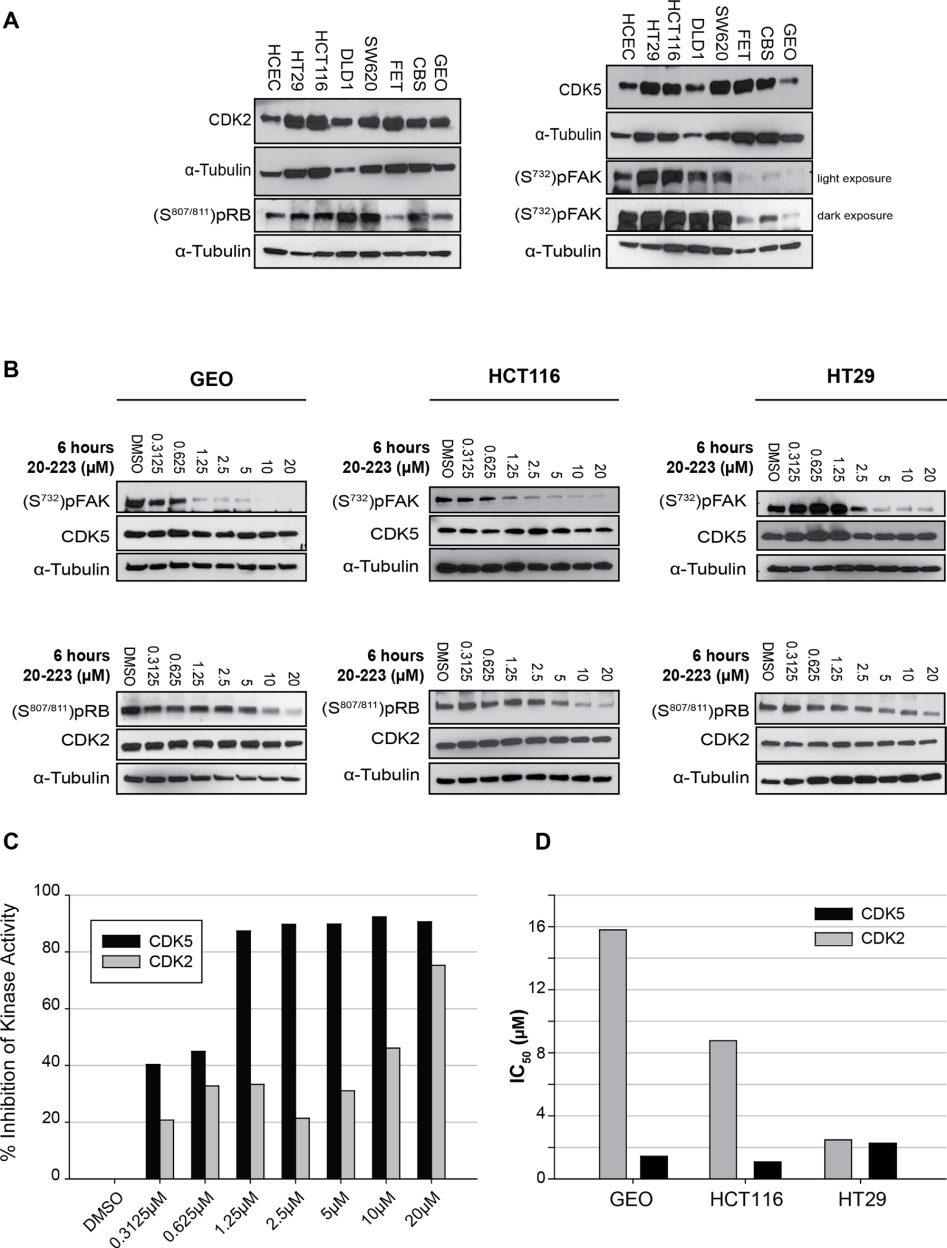
20-223 inhibits the kinase activity of CDK5 and CDK2 *in vitro*.

(**A**) Baseline expression of CDK2 and pRB (S807/811) (left), CDK5 and pFAK (S732) (right), in untreated CRC cells. (**B**) Representative western blots of target substrate pRB and pFAK phosphorylation levels in GEO (left), HCT116 (middle) and HT29 (right) cell lines after 6 hour incubation with 20-223. (**C**) Representative quantification of % inhibition of CDK2 and CDK5 kinase activity (based on substrate phosphorylation levels) in GEO cells found in Figure 3B. (**D**) Cell-based IC_50_ values generated from phosphorylation levels in Figure3B of CDK2 and CDK5 in three CRC cell lines.

