# Supplementary material for: Characterization of CDK(5) inhibitor, 20-223 (aka CP668863) for colorectal cancer therapy
Source: Oncotarget. 2017 Dec 28;9(4):5216–32. doi: 10.18632/oncotarget.23749 (PMC5797045; doi:10.18632/oncotarget.23749)
Supplement: Supplementary file 1 [file oncotarget-09-5216-s001.pdf]

Characterization of CDK(5) inhibitor, 20-223 (aka CP668863) for colorectal cancer therapy

SUPPLEMENTARY MATERIALS

STR profiling

All CRC cells used in this study have been previously reported (HCT116, HT29, DLD1, SW620) were validated prior to experimentation by determining their STR profile. Specimens were subjected to polymerase chain reaction (PCR) amplification using the Applied Biosystems AmpF*e*STR Identifier kit (University of Nebraska Medical Center – Human DNA Identification Laboratory). Upon receiving the DNA report STR analyses were cross-checked with ATCC. It is widely accepted that any cell line that is an 80% match to the ATCC online STR analyses can be considered valid (49, 50). FET, CBS, and GEO cell lines have been extensively used previously, however this is the initial report of their STR profile. FET, CBS, and GEO cell lines were also cross-checked with ATCC to verify no cross-contamination had occurred to these cell lines. (Supplementary Figure 9A).

20-223 synthesis

General methods

All reagents were purchased from commercial sources and were used without further purification. Flash chromatography was carried out on silica gel (200–400 mesh). Thin layer chromatography (TLC) were run on pre-coated EMD silica gel 60 F254 plates and observed under UV light at 254 nm and with basic potassium permanganate dip. Column chromatography was performed with silica gel (230-400 mesh, grade 60, Fisher scientific, USA). ^{1}H NMR (500 MHz) and ^{13}C NMR (125 MHz) spectra were recorded in chloroform-d or DMSO-d_6 on a Bruker instrument ($CDCl_3$ was 7.26 ppm for ^{1}H and 77.00 ppm for ^{13}C, DMSO was 2.5 ppm for ^{1}H and 39.00 ppm for ^{13}C). Proton and carbon chemical shifts were reported in ppm relative to the signal from residual solvent proton and carbon.

N-(5-cyclobutyl-1*H*-pyrazol-3-yl)-2-(naphthalen-2-yl)acetamide (CP-668863)

In a one neck round bottom flask placed, taken 5-cyclobutyl-1*H*-pyrazol-3-amine (0.7g, 5.10 mmol) in dichloromethane (20 mL) and added 4N KOH (2.28g, 40.8 mmol in 10 water). The reaction mixture was allowed to stir at room temperature followed by addition of Boc anhydride (1.17g, 5.35 mmol) in small batches. The reaction mixture was allowed to stir for 3h and reaction completion was monitored by thin layer chromatography (1:1 hexane: ethyl acetate). The reaction mixture was diluted in CH_2Cl_2, washed brine and dried with $MgSO_4$. The crude product was purified using silica gel column chromatography to yield *t*-butyl 3-amino-5-cyclobutyl-1H-pyrazole-1-carboxylate as off-white solid (1.06 g, 88%). ^{1}H-NMR ($CDCl_3$, 500 MHz) δ: 5.41 (s, 1H), 5.27 (br, 2H), 3.44 (p, J = 8.5 Hz, 1H), 2.25 (dtd, J = 3.1, 8.5, 11.6 Hz, 2H), 2.19 – 2.04 (m, 2H), 2.04 – 1.90 (m, 1H), 1.90 –1.78 (m, 1H), 1.61 (s, 9H), ^{13}C-NMR ($CDCl_3$, 125 MHz) δ: 161.8, 150.9, 140.4, 86.9, 85.4, 34.7, 28.7, 28.5, 18.9. To a solution of 2-(naphthalen-2-yl) acetic acid (2.0g, 10.74 mmol) in 50 mL of dry CH_2Cl_2 at 0 °C was added oxalyl chloride (2.76mL, 32.32 mmol). Catalytic amount of DMF (50 µL) was added and reaction mixture was allowed to warm to room temperature for 16h. The completion of the reaction was monitored by NMR spectroscopy by quenching a small aliquot in methanol. After completion of the reaction, reaction mixture was concentrated to dryness and the white solid was used in next step without further purification. To a clear solution of *t*-butyl 5-amino-3-cyclobutyl-1H-pyrazole-1-carboxylate (0.8g, 3.37 mmol) in dry CH_2Cl_2 (30 ml) at 0 °C was added diisopropylethylamine (1.2mL, 6.74 mmol). The reaction mixture was stirred at 0 °C for 10 min under argon atmosphere followed by addition of a solution of 2-(naphthalen-2-yl)acetyl chloride (0.9g, 4.38 mmol) in CH_2Cl_2 (10 mL) The reaction mixture was slowly warmed to room temperature and stirred for 3h. The progress of the reaction was monitored by thin layer chromatography. Following completion, the reaction mixture was quenched with brine and extracted with CH_2Cl_2, dried under $MgSO_4$ and concentrated to yield crude solid which was purified by silica gel column chromatography using hexane:ethyl acetate eluent (1.2g, 88%). ^{1}H-NMR ($CDCl_3$, 500 MHz) δ: 10.31 (s, 1H), 7.88 – 7.88 (m, 4H), 7.58 – 7.44 (m, 3H), 6.86 (s, 1H), 3.93 (s, 1H), 3.56 – 3.49 (m, 1H), 2.33 – 2.15 (m, 4H), 2.04 – 1.87 (m, 2H), 1.52 (s, 9H). To a solution of *t*-butyl 3-cyclobutyl-5-(2-napthalen-2-yl) acetamido)-1*H*-pyrazole-1-carboxylate (0.8g, 1.97 mmol) in CH_2Cl_2 (200 mL) at 0 °C was added, a dropwise solution of trifluoroacetic acid (20 mL). Reaction mixture was stirred at 0 °C for 1h. After completion of the reaction, the reaction mixture was concentrated under vacuum. The residue was further quenched with saturated sodium carbonate solution and extracted in CH_2Cl_2. The organic layer was washed with brine, dried under $MgSO_4$ and concentrated under vacuum. The crude solid was

repeatedly washed with 1:1 mixture of CH_2Cl_2 and hexane to yield CP668863 (0.51g, 84%) as white solid.^{1}H-NMR (CDCl3, 500 MHz) δ: ^{1}H NMR (500 MHz, DMSO-d_6) δ 12.05 (s, 1H), 10.57 (s, 1H), 7.87 (d, *J* = 3.7 Hz, 3H), 7.81 (s, 1H), 7.48 (s, 3H), 6.32 (s, 1H), 3.76 (s, 2H), 3.51 – 3.36 (m, 1H), 2.23 (m, 2H), 2.07 (m, 2H), 1.92 (q, J = 9.1, 9.7 Hz, 1H), 1.86 – 1.71 (m, 1H). ^{13}C NMR (125 MHz, DMSO-d_6) δ 168.49, 147.81, 134.41, 133.47, 132.30, 128.14, 128.12, 127.97, 127.89, 127.82, 126.58, 126.04, 93.91, 43.13, 31.54, 29.42, 18.58. HRMS calculated for $C_{19}H_{19}N_3O$ m/z 305.1528, found mass: 306.1627 (M+H).

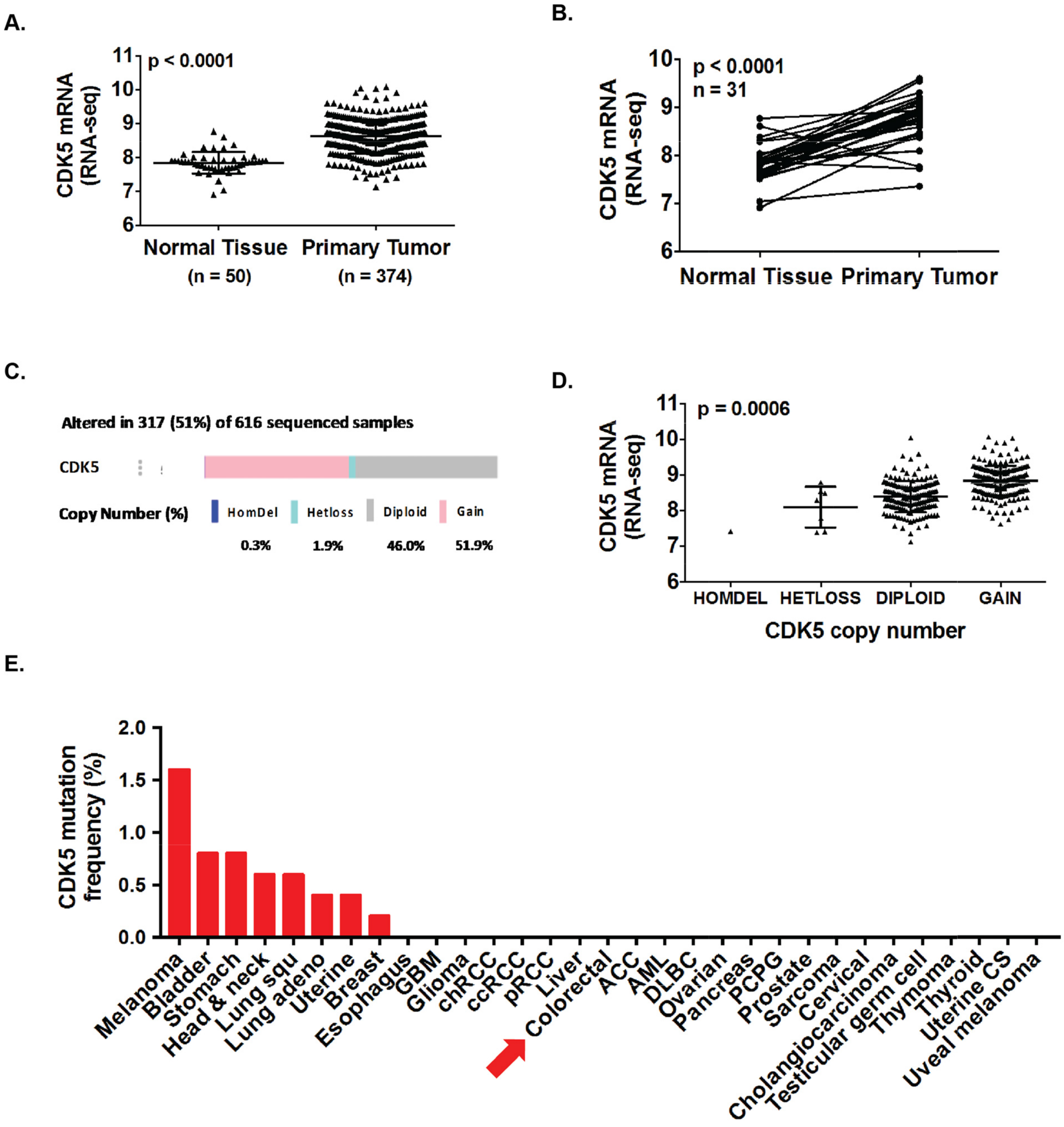

Supplementary Figure 1: CDK5 expression in TCGA patient samples. **(A)** CDK5 mRNA expression in normal CRC tissue (n=50) and primary tumor tissue (n=374). **(B)** Paired analysis of CDK5 mRNA expression of CRC patient (n=31) normal tissue and corresponding tumor samples. **(C)** CDK5 copy number status in 616 CRC samples. Shows the % of homozygous deletion (dark blue – 0.3%), heterozygous loss (light blue – 1.9%), diploid (grey - 46%), and copy number gain (pink – 51.9%) of CDK5 in CRC patients. **(D)** CDK5 mRNA expression compared to CDK5 copy number. **(E)** CDK5 mutation frequency across TCGA cancers. Arrow indicates CRC.

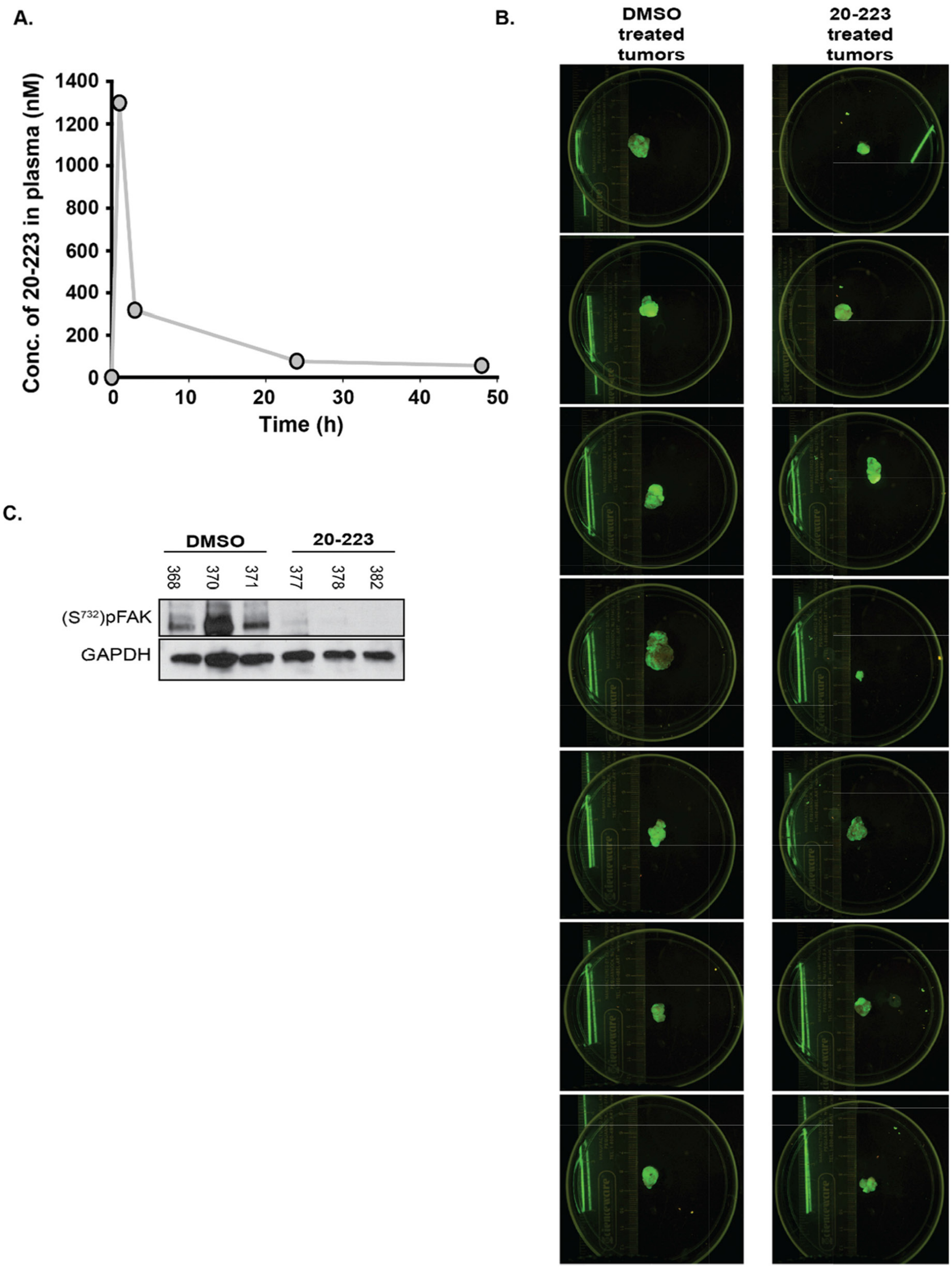

Supplementary Figure 2: *In vivo* analyses of 20-223 in a CRC xenograft model. **(A)** PK studies after mice were dosed with 8mg/kg 20-223. **(B)** Tumor images from DMSO and 20-223 treated mice. **(C)** pFAK (S732) levels from DMSO and 20-223 tumor lysates.

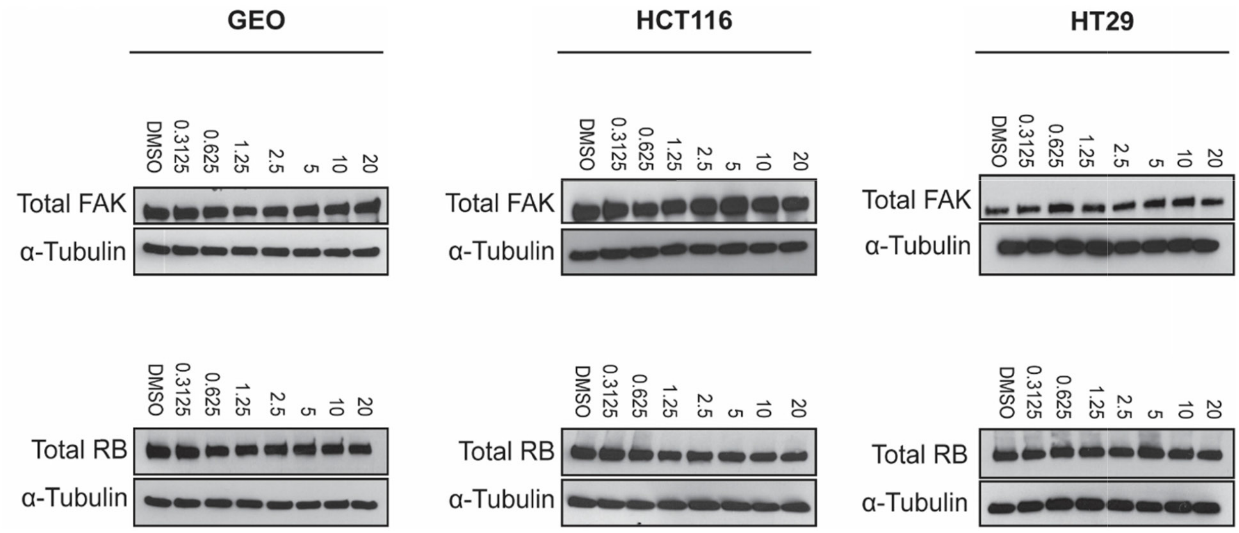

Supplementary Figure 3: Western blot analyses of total RB or FAK levels after GEO (left), HCT116 (middle), and HT29 (right) cells were treated with varying concentration of 20-223 for 6 hours.

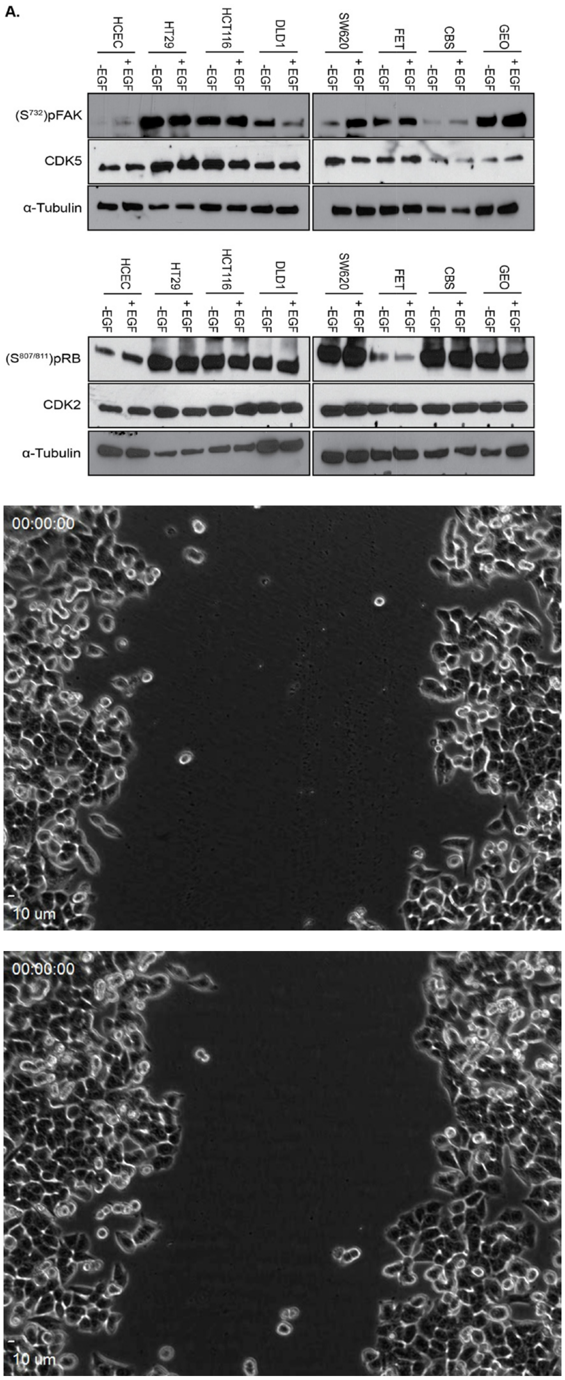

Supplementary Figure 4: EGF stimulation and live cell imaging videos of wound scratch assays. **(A)** Western blot analyses of CRC cells treated -/+ 100ng EGF. **(B & C)** Still images were taken every 15 minutes over 36 hour period (only 24 hours were considered for migration purposes to exclude any proliferative effects) and merged into a video. HCT116 cells were treated with (B) 100ng EGF + DMSO or (C) EGF + 1.5µM 20-223.

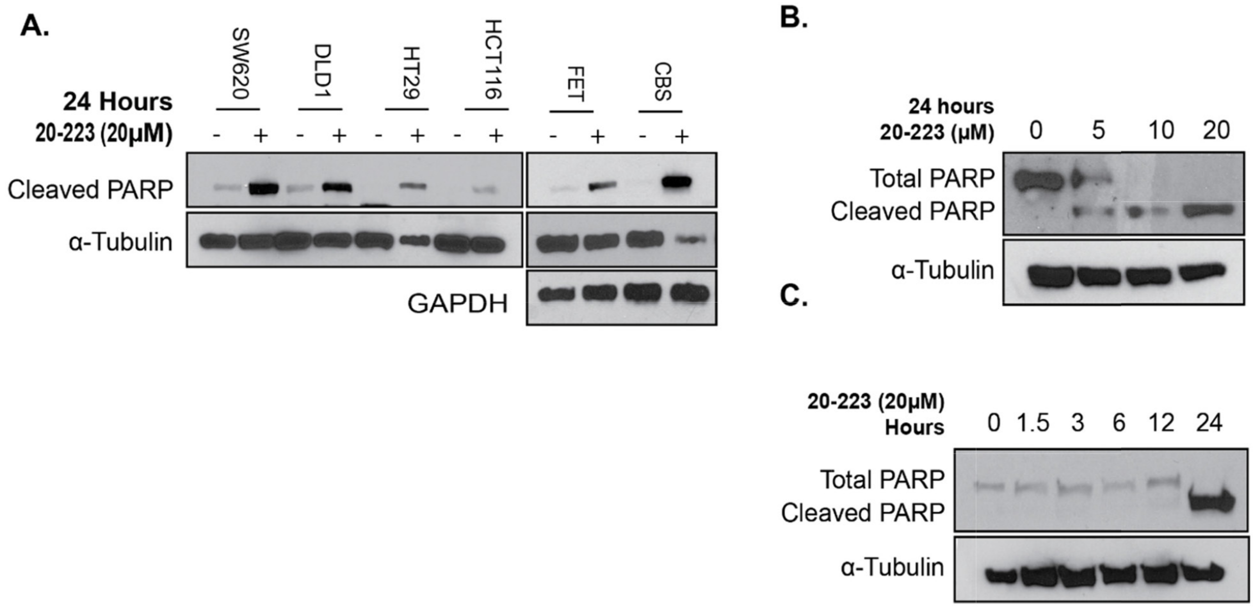

Supplementary Figure 5: Effect of 20-223 on cell death. **(A)** PARP cleavage in a panel of CRC cells treated with 20 μM of 20-223 for 24 hours. **(B)** Dose-response studies examined after 24 hour treatment with 20-223 in GEO cells. **(C)** Time-course studies to examine PARP cleavage after treatment of GEO cells with 20μM 20-223.

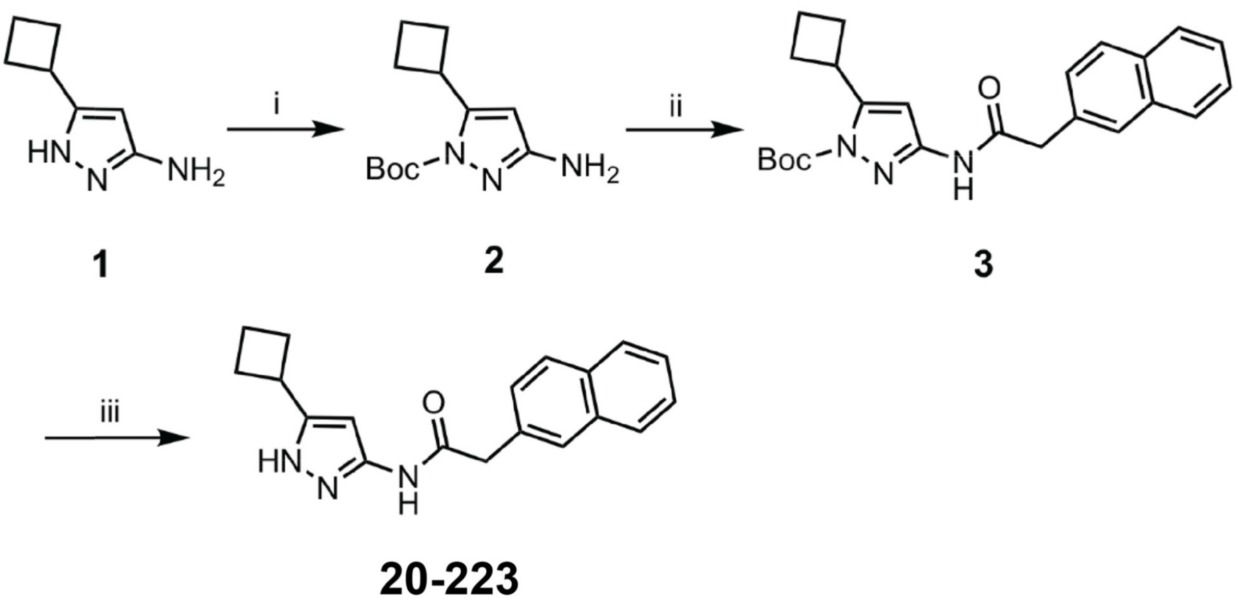

Supplementary Figure 6: Synthesis of 20-223. **(i)** $(BOC)_2O$, KOH, DCM:H_2O (1:1), RT, (88%); **(ii)** 2-(naphthalen-2-yl)acetyl chloride, DIPEA, DCM, 0 °C, (88%); **(iii)** CF_3CO_2H, RT, DCM, (84%).

A.

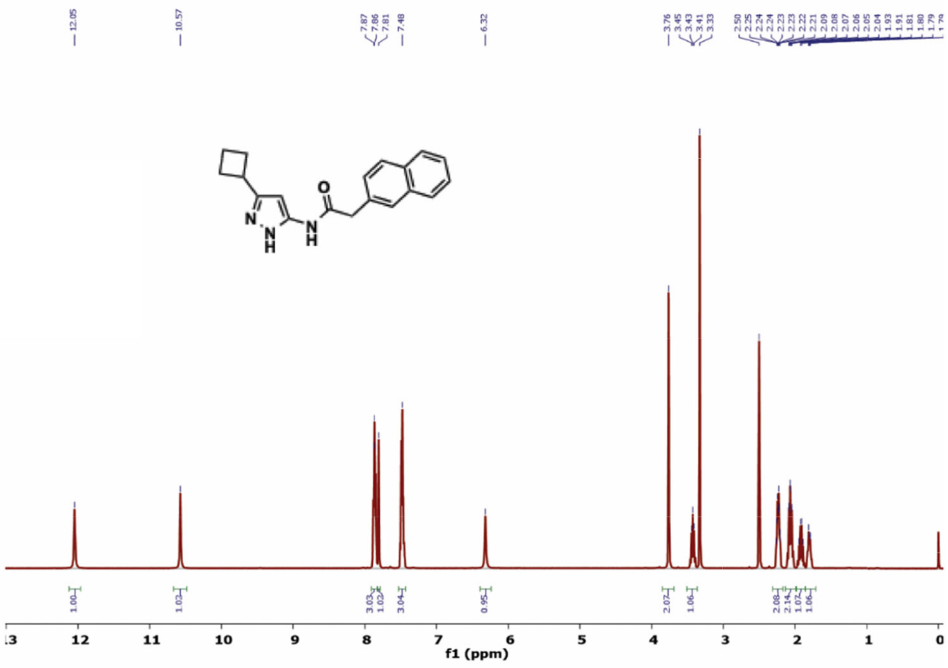

B.

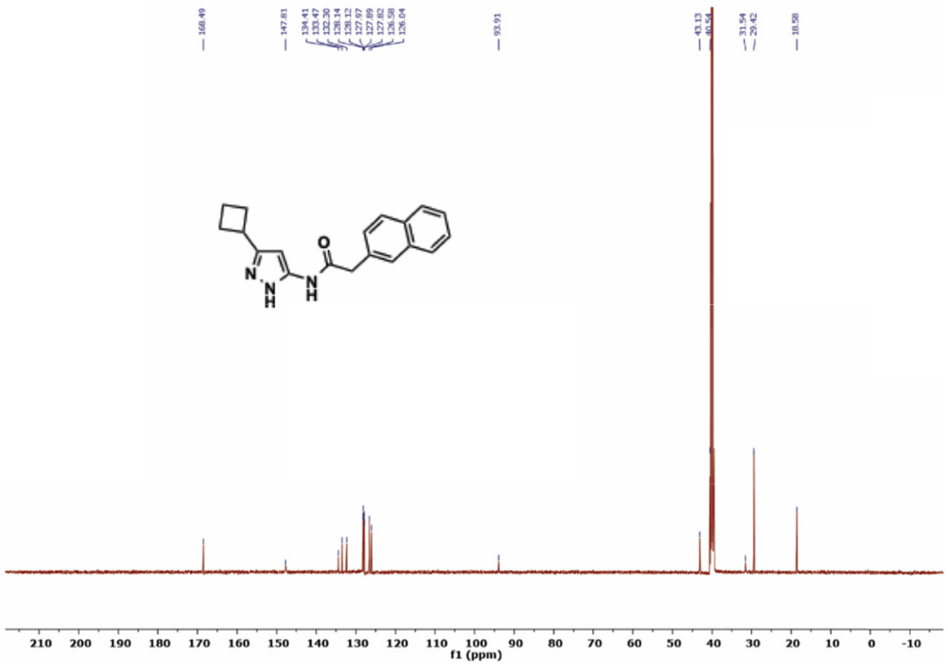

Supplementary Figure 7: Proton (top) and carbon (bottom) NMR confirmation of the 20-223 structure.

A. HRMS

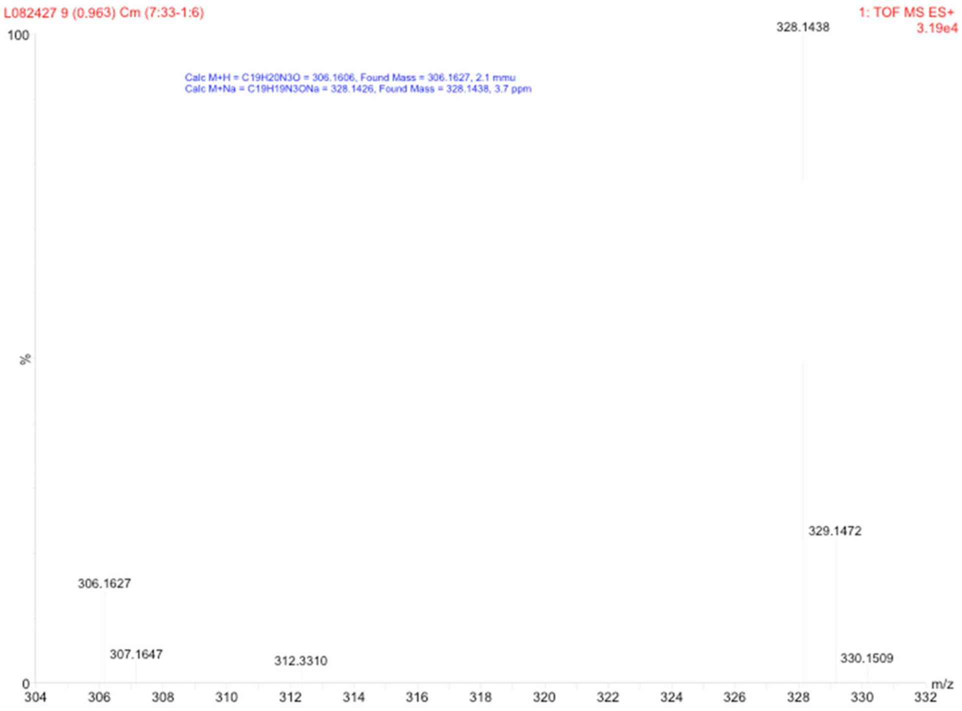

B. Analytical HPLC

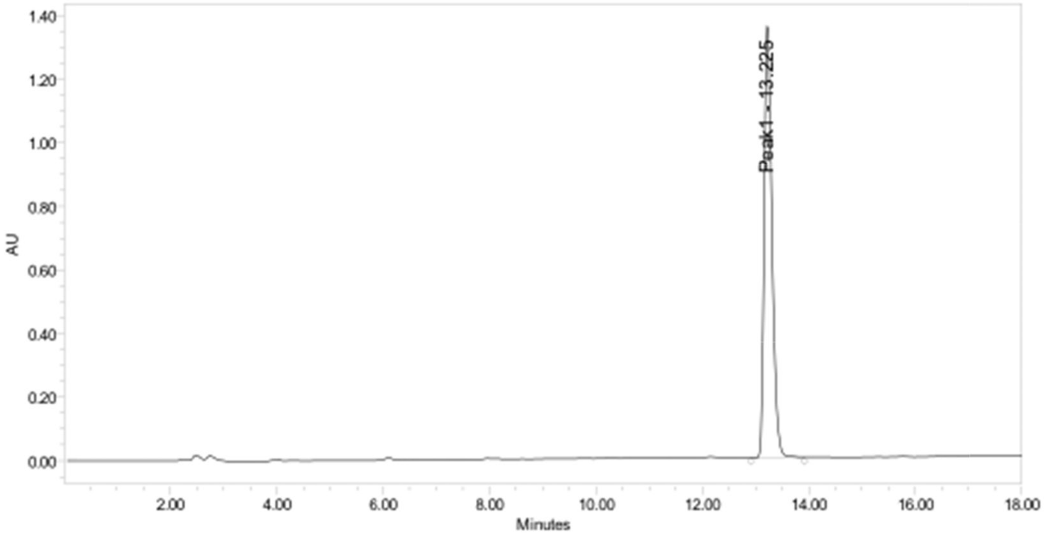

	Peak Name	RT	Area	% Area	Height
1	Peak1	13.225	13702758	100.00	1367578

Supplementary Figure 8: **(A)** HRMS and **(B)** Analytical HPLC of 20-223.

A.

Genetic Locus	HCT116	HT29	DLD1	SW620	FET*	CBS*	GEO*
D7S820	10,11,12	10	10,12	8,9	8,11	8,12	10,11,11.1
CSF1PO	7,10	11,12	11,12	13,14	12	12	13
THO1	8,9	6,9	7,9.3	8	7	8	8
D13S317	10,11,12,13	11,12	8,11	12	11,12	11,12	8,12
D16S539	11,12,13,14	11,12	12,13	9,13	12	9,11	12
vWA	17,18,21,22,23	17,19	18,19	16	16,17	16,17	15
TPOX	8	8,9	8,11	11	8	10,11	8
Amelogenin	X	X	XY	X	X	X	X
D5S818	10,11	11,12	13	13	13,14	12	12
% Match to ATCC database	89%	100%	88%	100%			

*first time STR profile characterization in previously used cell lines

B.

Antibody	Company	Catalog #	Lot #	Dilution of 1* Antibody	Source
α-Tubulin	Cell Signaling	13499	10	1 : 10,000	Mouse
CDK2	Cell Signaling	2546	6	1 : 2,000	Rabbit
CDK5	Cell Signaling	2506	2	1 : 2,000	Rabbit
CDK9	Cell Signaling	2316	5	1 : 2,000	Rabbit
pRB (S807/811)	Cell Signaling	9308	12	1 : 2,000	Rabbit
pFAK (S732)	Abcam	ab4792	GR2406125	1 : 2,000	Rabbit
pRPB1 (S2)	Cell Signaling	13499	1	1 : 5,000	Rabbit
total RB	Cell Signaling	9309	9	1 : 2,000	Mouse
total FAK	Cell Signaling	3285	9	1 : 2,000	Rabbit
total RPB1	Cell Signaling	2629	3	1 : 5,000	Mouse
total PARP	Cell Signaling	9542	13	1 : 10,000	Rabbit
cleaved PARP	Cell Signaling	9541	15	1 : 10,000	Rabbit
GAPDH	SantaCruz	sc-25778	C2415	1 : 4,000	Rabbit
BCL-XL	Cell Signaling	2762	7	1 : 2,000	Rabbit
MCL-1	Cell Signaling	5453	4	1 : 2,000	Rabbit

Supplementary Figure 9: Validation of cell lines and antibodies used. **(A)** Cell line validation after STR profile analyses ross-checked with ATCC. >80% is considered valid. **(B)** Antibody information and usage. Validation provided by datasheets supplied by respective companies.
